# Meta-analysis of the correlation between pulmonary hypertension and echocardiographic parameters in patients with chronic kidney disease

**DOI:** 10.7717/peerj.17245

**Published:** 2024-04-19

**Authors:** Jiahui Jin, Wen Hao, Deqiong Xie

**Affiliations:** 1 School of Medicine and Life Sciences, Chengdu University of Traditional Chinese Medicine, Chengdu, China; 2 Department of Nephrology, Yibin Second People’s Hospital, Yibin, China

**Keywords:** Chronic kidney disease, Pulmonary hypertension, Echocardiography, Meta-analysis

## Abstract

**Objective:**

To investigate the correlation between pulmonary hypertension (PH) and echocardiographic parameters in patients with chronic kidney disease (CKD).

**Methods:**

PubMed, Embase, Web of Science, Cochrane, VIP, CNKI, and Wanfang databases were systematically searched for articles published from inception to 19 May 2023. Study quality was estimated using the Quality Assessment of Case-Control Studies tool. Forest plots were drawn using R language software. The “metacor” function in the “meta” package was utilized for meta-analysis of the *r*-values and their standard errors. Heterogeneity and sensitivity analyses were carried out, with the main outcomes as *r*-value, *p*-value, and *I^2^* value.

**Results:**

Eleven studies were included, with 1,809 CKD patients. The correlations between 12 echocardiographic parameters and PH were analyzed. Except for FS and LVEF which were negatively correlated with CKD-PH, the other 10 parameters were positively correlated with CKD-PH. Among them, LA was highly correlated with CKD-PH (0.70 < *r* < 0.89); LVDD, RA, RV, LVMI, and LVDS were moderately correlated with CKD-PH (0.40 < *r* < 0.69); while PA, IVS, LVPW, SV, FS, and LVEF were lowly correlated with CKD-PH (0.20 < *r* < 0.39). The synthesized estimates were stable against heterogeneity.

**Conclusion:**

CKD-PH patients may have large cardiac chambers, thickened septal tissue on both sides of the chambers, reduced pulmonary artery flow rates, and decreased left ventricular function.

## Introduction

By the year 2040, chronic kidney disease (CKD) is anticipated to be the fifth leading cause of death globally. In adults aged ≥20 years, the prevalence of CKD is expected to reach as high as 11.1% (Kuro-o et al., 1997). It is considered one of the major public health concerns worldwide due to its substantial impact on society, including high treatment costs, multiple complications, and poor prognosis (Jager et al., 2019). Cardiovascular complications are more prevalent and serious in CKD patients than in the general population (Thomas et al., 2017). These complications are the leading factors contributing to mortality in patients with end-stage renal disease (ESRD) (Walther et al., 2020). CKD patients are most commonly afflicted by cardiovascular disease (CVD), which is identified as the most prevalent cause of morbidity and mortality in this population (Heine et al., 2020). Pulmonary hypertension (PH), a prevalent and serious complication in CKD patients, is known to affect up to 50% of ESRD patients (Devasahayam et al., 2020). The incidence of PH increases with disease progression, ranging from 9–39% in CKD patients at stage 5, which is 2–3 times higher than that in patients at stage 2–3. Additionally, the incidence of PH in patients undergoing hemodialysis is as high as 58.9% (Bai, Cui & Liu, 2022). In ESRD patients, PH has been established as an independent predictor of all-cause mortality and CVD (Bai, Cui & Liu, 2022). Moreover, the presence of PH is associated with an increased incidence of cardiovascular complications. A recent meta-analysis revealed that PH was associated with a higher risk of death and CVD in patients with CKD and ESRD (Tang et al., 2018). Nevertheless, the connection between PH and CKD remains unclear, and the presence of traditional risk factors such as hypertension, hyperlipidemia, and diabetes mellitus does not fully explain this association. Therefore, CKD-PH perhaps has a unique pathogenesis (Page et al., 2021).

According to the latest ERS/ESC classification in 2022, CKD-induced PH is classified as type V (PH due to unknown mechanisms) (Humbert et al., 2022). Currently, the gold standard for PH diagnosis is the right heart catheterization using floating catheters (Meinel et al., 2020). However, the extensive application of this diagnostic procedure in clinical practice is challenging due to its high cost, invasive nature, potential risks, and the requirement for technical support from experienced clinicians. Echocardiography is commonly considered the primary method for screening PH due to its affordability and non-invasive nature, despite its limitations in accurately estimating parameters (Tiengo, Fadini & Avogaro, 2008). PH is assessed based on tricuspid regurgitant velocity (TRV) and right atrial pressures (RAP) through echocardiography using the simplified Bernoulli’s equation formula: PH = 4 × (TRV)^2^ + RAP (Poch & Mandel, 2021).

The influence of CKD and PH on cardiac morphology is profound and has been extensively investigated. Nevertheless, the relationship between echocardiographic parameters and CKD-PH has not yet been summarized. Therefore, this article synthesizes the available evidence to unveil the relationship between PH and echocardiographic indices in CKD patients.

## Methods

### Registration

We mistakenly filled in the “time period” for May 4th during registration, and the correct time should be May 19th. However, when we later discovered it, we were no longer able to modify it on PROSPERO. Please refer to the search time in the article for accuracy. Moreover, to check if there are some new eligible articles, we updated the literature search on 1 December 2023, and nothing new was found. This update was described in our revised manuscript, too. This article followed the PRISMA statement (Mills et al., 2015). The review program and records were available online through PROSPERO (#CRD42023407420) (Jin, Xie & Hao, 2023).

### Article retrieval

PubMed, Embase, Web of Science, Cochrane, VIP, CNKI, and Wanfang databases were searched using the main search terms “chronic kidney disease” and “pulmonary hypertension”. The search strategy was developed under the guidance of an experienced researcher (Wen Hao) and detailed in the Supplemental Materials. The searches were conducted firstly on 19 May 2023 and updated to 1 December 2023 before submission, with no language or other restrictions. References in the included articles were further manually searched.

### Article screening criteria

Article screening was carried out independently by two researchers (Jiahui Jin, Deqiong Xie) and any disagreements were resolved by a third experienced researcher (Wen Hao). After eliminating duplicates, the titles and abstracts of the remaining articles were reviewed to determine the initial article inclusion, and then the final inclusion was determined by reading the full text. Inclusion criteria were as follows: (1) patients with CKD; (2) receiving echocardiography and with extractable data; (3) conducting correlation analysis between PH and other echocardiographic parameters. The articles were excluded for the following reasons: (1) reviews, case reports, conference proceedings, animal and cellular experiments, and unrelated literature; (2) for different studies on the same study population, only the latest study was included, and others were excluded; (3) unclear diagnostic criteria for cases; (4) duplicate articles; and (5) unavailability of data for statistical analysis.

### Risk of bias assessment

The risk of bias was assessed independently by two researchers using the NIH Quality Assessment of Case-Control Studies (National Heart, Lung, and Blood Institute, 2021), and the results were combined by a third researcher. The tool consisted of 12 aspects: (1) Definition of study purpose; (2) definition of the population; (3) sample size justification; (4) consistency of included population; (5) strict implementation of standards for all participants; (6) clear definition of disease; (7) case and control groups were randomly selected; (8) with a control group; (9) researchers know exposure and occurrence risks in advance; (10) the factors associated with exposure/risk measurement of participants are strictly implemented; (11) researchers do not know the disease status of the participants; (12) accurate calculation of numerical variables. The assessment results of each aspect included YES, NO, and NR (including three options: CD: cannot determine, NA: not applicable, NR: not report), with YES scored as 1 point and NO and NR scored 0. Based on the total scores, 0–4 represented poor (low quality), 5–8 fair (medium quality), and 9–12 good (high quality).

### Data extraction

Data were extracted independently by two researchers according to the data table prepared in advance and merged by the third one. The extracted data included (1) basic study information: first author, year of publication, and region or country; (2) patient characteristics: sample size, age, gender, and creatinine value; and (3) outcome index: correlation between each echocardiographic parameter and PH.

### Statistical analyses

Forest plots were drawn using R language software 4.2.3 (R Core Team, 2023). The “metacor” function in the “meta” package was utilized for meta-analysis of the *r*-values and their standard errors. First, heterogeneity tests were performed. If *I*^*2*^> 50% and *p* < 0.05, the random-effect models (REM) were used, otherwise, the fixed-effect models (FEM) were used. Sensitivity analysis was performed for REM, including subgroup analysis and leave-one-out method The result was statistically different with *p* < 0.05, otherwise, it was not statistically different. If there were more than 10 studies, the publication bias was assessed using Egger’s tests, with *p* ≥ 0.05 indicating there was no significant publication bias, otherwise, there was significant publication bias. The results of the meta-analyses were presented using forest plots.

## Results

### Literature retrieval results

**Figure 1:**
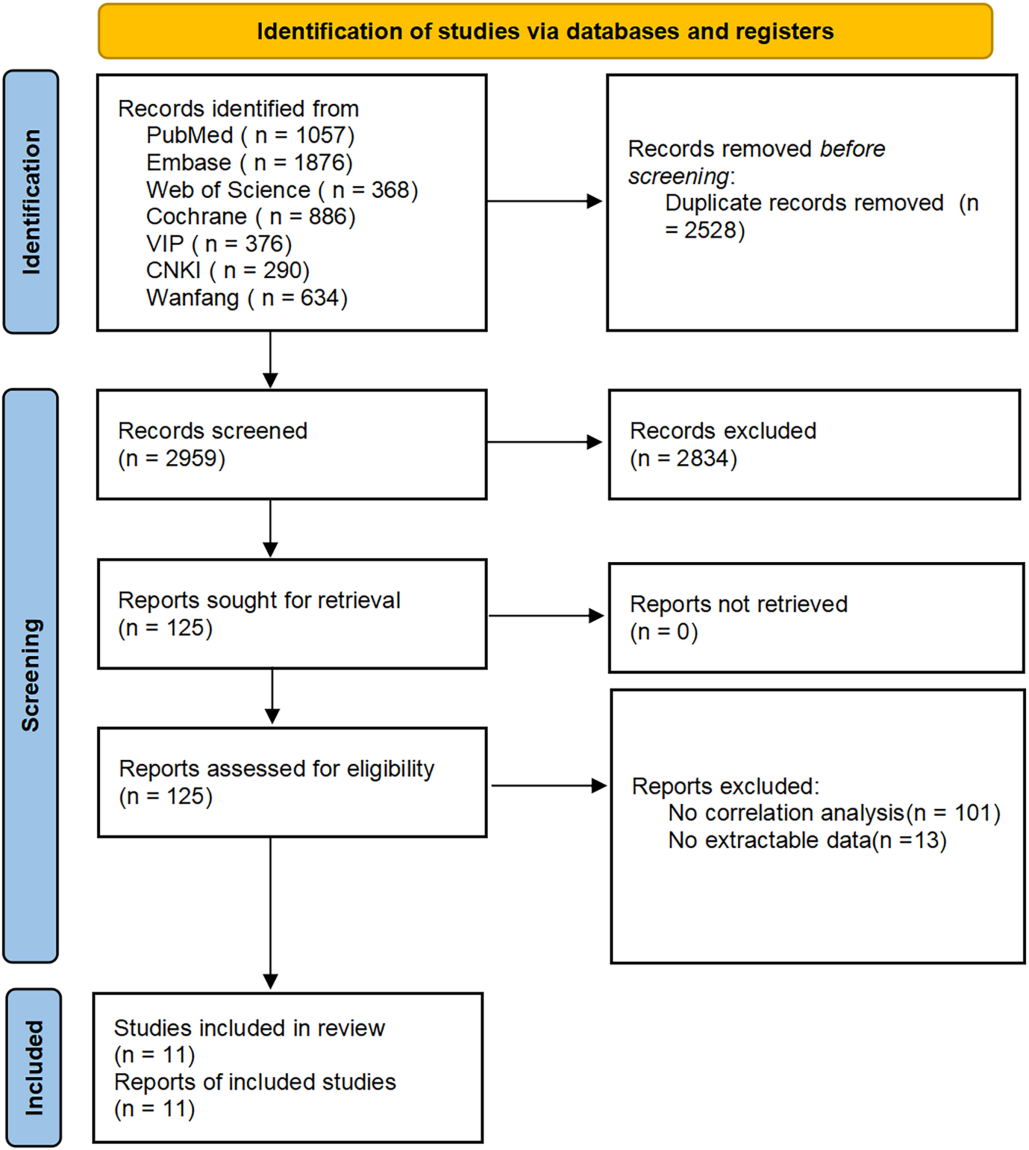
Flowchart of article retrieval and screening.

In 3,327 articles were initially searched, of which 368 duplicates were excluded; 2,083 articles that did not meet the inclusion criteria were excluded after reading the abstracts; 73 animal studies, 116 conference proceedings, 424 registrations or protocols, 96 reviews, and 42 meta-analyses were also excluded. After reading the full text, articles on other related factors of PH such as inflammatory indicators, parathyroid hormone, creatinine, glomerular filtration rate, and other renal function indicators were excluded. Additionally, articles with unavailable full text and without correlation analyses were excluded. Articles that did not list the detailed correlation between echocardiographic parameters and PH in CKD patients were excluded due to unavailable data. Finally, 11 articles were included, with 1,809 patients. 12 parameters were evaluated in the echocardiography, including LA (left atrium), LVDD (left ventricular end-diastolic diameter), RA (right atrium), RV (right ventricle), PA (pulmonary artery diameter), IVS (interventricular septal thickness), LVPW (left ventricular posterior wall thickness), FS (left ventricular short axis systole rate), LVEF (left ventricular ejection fraction), SV (left ventricular per beat volume), LVMI (left ventricular mass index), and LVDS (left ventricular end-systolic diameter). The literature acquisition process is shown in Fig. 1.

### Risk of bias

The risk of bias was evaluated in 11 articles and was all rated as good, indicating that the overall risk of bias was low, possibly because researchers knew the disease status of the participants. None of the studies blinded the researchers to the disease status of the patients. All other aspects met the NIH-QAT requirements for a well-designed case-control study. Table S1 displays the details of the evaluation results.

### Characteristics of the included studies

**Figure 2:**
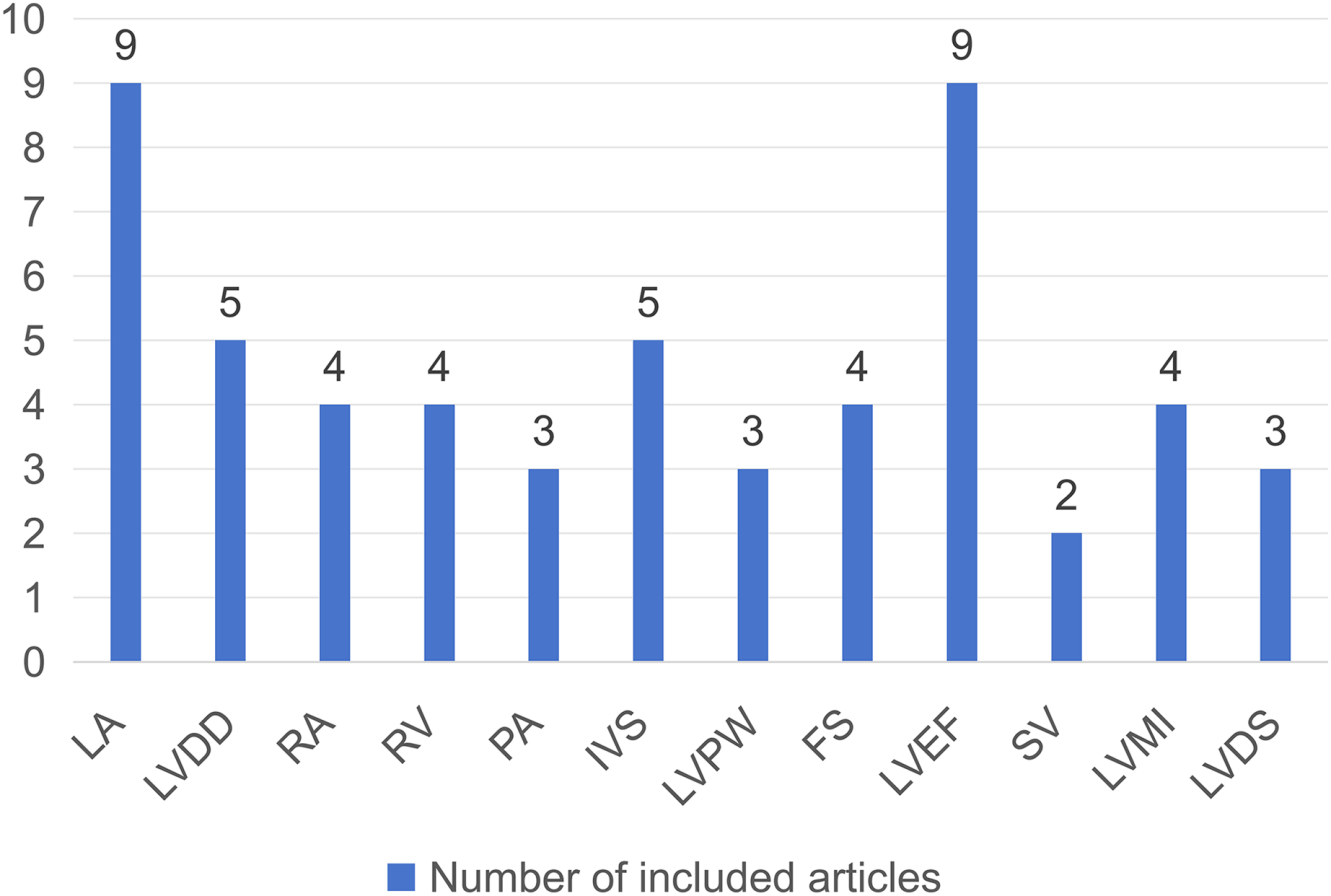
Bar chart incorporating echocardiographic parameters and number of related articles.

The 11 studies (including 1,809 CKD patients, with both males and females) were all conducted in Asian countries and published between 2009 and 2022. The inclusion indicator for all participants was creatinine value >178 umol/L or glomerular filtration rate <60 ml/min. Information on the basic characteristics of the included studies is presented in Table 1. Among them, nine articles investigated the relationship between LA and PH in CKD patients, five articles on LVDD, four articles on RA, four articles on RV, three articles on PA, five articles on IVS, three articles on LVPW, four articles on FS, nine articles on LVEF, two articles on SV, four articles on LVMI, and three articles on LVDS. The number of articles involved in each echocardiographic parameter is shown in Fig. 2.

**Table 1:** Basic feature information included in the study.

Study	Population	Age (mean, SD)	Gender (female, male)	Creatinine (mean, SD)	GFR (mean, SD)	LVEF (mean, SD)	sPAP (mean, SD)	TRV (mean, SD)	∆P (mean, SD)	Quality rating
Ding et al. (2022)	163	67.35, 10.42	86, 70	759.61, 303.42	NR	0.62, 0.09	27.46, 3.97	235.58, 22.72	22.54, 4.23	Good
Yang & Bao (2014)	128	53.59, 17.52	66, 62	NR	66.49, 32.60	0.64, 0.06	32.37, 8.55	NR	NR	Good
He et al. (2015)	136	52.00, 16.38	56, 80	NR	NR	0.63, 0.10	NR	NR	NR	Good
Zhang et al. (2020)	283	52.63, 14.28	107, 176	818.90, 295.18	NR	0.60, 0.10	NR	276.31, 53.67	31.24, 13.17	Good
Yang & Bao (2012)	397	52.17, 18.56	189, 208	NR	68.32, 32.47	0.63, 0.06	31.65, 7.56	NR	NR	Good
Lv et al. (2019)	94	60.65, 13.16	42, 52	266.56, 162.21	25.00, 12.08	0.59, 0.07	35.27, 13.26	NR	NR	Good
Feng & Ren (2020)	144	52.70, 14.60	61, 83	703.71, 78.49	NR	0.65, 0.08	38.72, 12.41	NR	NR	Good
Wang et al. (2014a)	76	45.90, 11.90	35, 41	NR	NR	0.61, 0.12	NR	651.32, 185.83	NR	Good
Wang et al. (2014b)	136	NR	NR	NR	NR	0.63, 0.10	NR	NR	NR	Good
Shen et al. (2021)	219	60.62, 14.66	99, 120	663.25, 220.63	NR	0.60, 0.10	38.27, 11.94	NR	NR	Good
Gao et al. (2021)	33	46.00, 14.61	14, 19	859.06, 260.85	NR	0.64, 0.12	24.76, 20.63	NR	NR	Good

**Note:**

mean, average value; SD, standard deviation; NR, not mentioned; GFR, glomerular filtration rate; LVEF, left ventricular ejection fraction; sPAP, pulmonary artery pressure; TRV, tricuspid regurgitation velocity; ∆P, tricuspid valve pressure difference.

### Results of statistical analysis

In the 11 articles, 12 parameters associated with PH in CKD patients were involved, including three categories: left heart parameters (LA, LVDD, LVPW, FS, LVEF, SV, LVMI, and LVDS), right heart parameters (RA and RV), and other parameters (IVS and PA). Except for FS and LVEF, which were negatively correlated with PH in CKD patients, the other 10 parameters were positively correlated. The parameters that were highly correlated with PH in CKD patients were LA, those moderately correlated with PH in CKD patients were LVDD, RA, RV, LVMI, and LVDS, and those lowly correlated with PH in CKD patients were PA, IVS, LVPW, SV, FS, and LVEF. Detailed information on each of the 12 parameters is provided in Table 2. The forest plots of correlation analysis are displayed in the Supplemental Materials.

**Table 2:** Statistical analysis of echocardiographic parameters related to PH in CKD patients.

Group	Index	Sample size		Heterogeneity	Pooled estimation	Sensitivity analysis	Forest plot
Left heart	LA	nine studies, 1,496 patients		*I*^*2*^= 98%, *p* < 0.01	*r* (95% CI) = 0.74 [0.47–0.88], *p* < 0.01	Stable	Figs. S1, S13
	LVDD	five studies, 862 patients		*I*^*2*^= 76%, *p* < 0.01	*r* (95% CI) = 0.45 [0.34–0.55], *p* < 0.01	Stable	Figs. S2, S14
	LVPW	three studies, 843 patients		*I*^*2*^= 72%, *p* < 0.01	*r* (95% CI) = 0.24 [0.16–0.36], *p* = 0.03	Stable	Figs. S3, S17
	FS	four studies, 676 patients		*I*^*2*^= 39%, *p* < 0.01	*r* (95% CI) = −0.30 [−0.36 to −0.23], *p* = 0.18	–	Fig. S4
	LVEF	nine studies, 1,284 patients		*I*^*2*^= 64%, *p* < 0.01	*r* (95% CI) = −0.31 [−0.39 to −0.22], *p* = 0.05	Stable	Figs. S5, S19
	SV	two studies, 446 patients		*I*^*2*^= 29%, *p* < 0.01	*r* (95% CI) = 0.20 [0.11–0.29], *p* = 0.23	–	Fig. S6
	LVMI	four studies, 846 patients		*I*^*2*^= 93%, *p* < 0.01	*r* (95% CI) = 0.45 [0.18–0.66], *p* < 0.01	Stable	Figs. S7, S18
	LVDS	three studies, 263 patients		*I*^*2*^= 53%, *p* < 0.01	*r* (95% CI) = 0.40 [0.30–0.50], *p* = 0.12	–	Fig. S8
Right heart	RA	four studies, 718 patients		*I*^*2*^= 40%, *p* < 0.01	*r* (95% CI) = 0.51 [0.45–0.56], *p* = 0.17	–	Fig. S9
	RV	four studies, 718 patients		*I*^*2*^= 87%, *p* < 0.01	*r* (95% CI) = 0.65 [0.51–0.76], *p* < 0.01	Stable	Figs. S10, S15
Other	IVS	five studies, 1,115 patients		*I*^*2*^= 97%, *p* = 0.03	*r* (95% CI) = 0.33 [0.04–0.56], *p* < 0.01	Stable	Figs. S11, S16
	PA	three studies, 582 patients		*I*^*2*^= 0%, *p* < 0.01	*r* (95% CI) = 0.36 [0.29–0.43], *p* = 0.39	–	Fig. S12

**Note:**

LA, left atrium; LVDD, left ventricular end diastolic diameter; LVPW, thickness of the posterior wall of the left ventricle; FS, left ventricular short axis systolic rate; LVEF, left ventricular ejection fraction; SV, left ventricular stroke volume; LVMI, left ventricular mass index; LVDS, left ventricular end systolic diameter; RA, right atrium; RV, right ventricle; IVS, thickness of the interventricular septum; PA, inner diameter of the pulmonary artery.

Among left heart parameters, LA was highly correlated with PH [9 studies, 1,496 patients; *I*^*2*^= 98%, *p* < 0.01; REM; *r* (95% CI) = 0.74 [0.47–0.88], *p* < 0.01]. LVDD [five studies, 862 patients; *I*^*2*^= 76%, *p* < 0.01; REM; *r* (95% CI) = 0.45 [0.34–0.55], *p* < 0.01], LVMI [four studies, 846 patients; *I*^*2*^= 93%, *p* < 0.01; REM; *r* (95% CI) = 0.45 [0.18–0.66], *p* < 0.01], and LVDS [three studies, 263 patients; *I*^*2*^= 53%, *p* < 0.01; FEM; *r* (95% CI) = 0.40 [0.30–0.50], *p* = 0.12] were moderately correlated with PH. LVPW [3 studies, 843 patients; *I*^*2*^= 72%, *p* < 0.01; REM; *r* (95% CI) = 0.24 [0.11–0.36], *p* = 0.03], FS [four studies, 676 patients; *I*^*2*^= 39%, *p* < 0.01; FEM; *r* (95% CI) = −0.30 [−0.36 to −0.23], *p* = 0.18], LVEF [nine studies, 1,284 patients, *I*^*2*^= 64%, *p* < 0.01; REM; *r* (95% CI) = −0.31[−0.39 to −0.22], *p* = 0.05], and SV [two studies, 446 patients; *I*^*2*^= 29%, *p* < 0.01; FEM; *r* (95% CI) = 0.20 [0.11–0.29], *p* = 0.23] were lowly associated with PH.

Among right heart parameters, RA [four studies, 718 patients; *I*^*2*^= 40%, *p* < 0.01; FEM; *r* (95% CI) = 0.51 [0.45–0.56], *p* = 0.17] and RV [4 studies, 718 patients; *I*^*2*^= 87%, *p* < 0.01; REM; *r* (95% CI) = 0.65 [0.51–0.76], *p* < 0.01] were moderately associated with PH.

As for other parameters, IVS [five studies, 1115 patients; *I*^*2*^= 97%, *p* = 0.03; REM; *r* (95% CI) = 0.33 [0.04–0.56], *p* < 0.01] and PA parameters [three studies, 582 patients; *I*^*2*^= 0%, *p* < 0.01; FEM; *r* (95% CI) = 0.36 [0.29–0.43], *p* = 0.39] were lowly associated with PH.

Due to significant heterogeneity (*I*^*2*^> 50%, *p* < 0.05) in the results of LA, LVDD, RV, IVS, LVPW, LVMI, and LVEF, sensitivity analyses were performed using the leave-one-out method, which revealed that all synthesized estimates were stable against heterogeneity. The forest plot of sensitivity analysis is exhibited in the Supplemental Materials.

Finally, funnel plots were made for each of the 12 parameters associated with PH in CKD patients in the 11 included articles. The results of these funnel plots indicated no significant publication bias. However, because the number of relevant studies included for each parameter was less than 10, the results of Egger’s test were unreliable under such conditions. Therefore, we did not perform the test. The funnel plots of the correlation analyses are shown in the attachment.

## Discussion

This study discovered that CKD-PH may be associated with large cardiac chambers, thickened septal tissue on both sides of the chambers, reduced pulmonary artery flow rates, and LV dysfunction. CKD-PH is characterized by a complex interplay of factors that affect the heart. The unique therapeutic approaches for CKD, comorbidities, and shared factors with PH collectively affect the cardiac structure and functions, leading to the distinct echocardiographic manifestations of CKD-PH. Taken together, the enlarged left heart cavity may be attributed to increased blood pressure, fluid retention, and arteriovenous fistula (AVF). The enlarged right heart cavity is an intrinsic feature of PH, and it may share certain mechanisms with the changes in the left heart cavity. The thickened interventricular septum may be related to the increase in blood pressure.

CKD patients may have abnormalities of some substances including fibroblast growth factor23 (FGF23)-klotho protein (KLOTHO), asymmetric dimethylarginine (ADMA), and inflammatory factors due to renal failure. These abnormalities profoundly affect the circulatory system, leading to cardiac remodeling and PH. Abnormalities in the FGF23-KLOTHO axis, characterized by increased FGF23 and diminished KLOTHO levels, can lead to LV hypertrophy and increased mass (National Heart, Lung, and Blood Institute, 2021). Animal studies similarly substantiated the aforementioned results and confirmed the presence of cardiotoxicity (Faul et al., 2011). Both the FGF23-KLOTHO axis and parathyroid hormone are strongly associated with PH severity, but their roles in CKD-PH are not fully understood (Zhang et al., 2018). Plasma ADMA impairs the vasoprotective function of high-density lipoprotein, thereby enhancing the risk of coronary artery disease (Zewinger et al., 2017). Moreover, ADMA acts as an inhibitor of endogenous nitric oxide synthesis and is synthesized in significant amounts in CKD, resulting in its accumulation in the lungs (Devasahayam et al., 2020). ADMA has been suggested to be potentially correlated with CKD-PH (Bolignano et al., 2013). Levels of several inflammatory biomarkers such as interleukin-6 (IL-6) and tumor necrosis factor (TNF) are elevated in the context of CKD (Gilligan & Raphael, 2017; Yu et al., 2009). CKD-induced inflammation can initiate endothelial dysfunction, which disrupts the balance of vasomotor function and leads to abnormal pulmonary vasoconstriction and fibrosis (Edmonston & Sparks, 2020). Chronic inflammation is a key player in the pathogenesis of various cardiac structural changes, such as cardiomyopathy (Kakraba et al., 2023). It is implicated in the inflammatory state due to other diseases and in the microinflammatory state in CKD, thus exacerbating cardiac remodeling and promoting PH progression (von Siebenthal et al., 2016). Since the renal excretory function is gradually decompensated, CKD patients suffer from abnormalities in the internal environment, such as disturbances in calcium and phosphorus metabolism. The microinflammatory state of the body can lead to gradual myocardial fibrosis and even myocardial hypertrophy or other structural changes (Bonacina et al., 2016; Doni et al., 2015). The large accumulation of uremic toxins in the internal environment, including large molecules such as parathyroid hormone, and medium molecules such as peptides produced due to cellular metabolism disorders, acts on the heart and lead to cardiac damage (Valkenburg, Glorieux & Vanholder, 2021). Accumulation of small molecule substances guanidines and amines can inhibit platelet function and lead to pulmonary edema, increasing the burden on the cardiovascular system (Evans, Reddan & Szczech, 2004). These changes, in turn, contribute to PH.

The complications of fluid retention, obstructive sleep apnoea/hypopnoea syndrome (OSAHS), anemia, and hyperparathyroidism in CKD patients lead to both cardiac structural changes and PH. Echocardiography has revealed that patients with high heart disease frequently display different levels of thickening of the interventricular septum (Li & Jiamarideng, 2014). CKD patients commonly experience fluid retention and subsequent hypertension. Consequently, it is reasonable that CKD-PH patients exhibit similar manifestations in the septum as those observed in individuals with high heart disease (He & MacGregor, 2011). On the other hand, mechanisms such as uremic toxin-induced neuropathy or myopathy, altered chemosensitivity, and hypervolemia in CKD or ESRD increase the risk of developing sleep apnoea (Lin, Lurie & Lyons, 2020). As a result, OSAHS is common in patients with CKD and ESRD (Nicholl et al., 2012), potentially leading to recurrent hypoxemia and an imbalance of the sympathovagal system. Hypoxemia aggravates PH by directly promoting pulmonary vasoconstriction (Edmonston & Sparks, 2020) and causes concentric LV hypertrophy (Zoccali et al., 2001). In addition, CKD can give rise to anemia, and prolonged anemia leads to a sustained increase in cardiac output, which in turn results in cardiac remodeling (Eckardt, 2005) and hypoxia. Similar to OSAHS-induced PH, anemia can aggravate PH by indirectly inducing pulmonary vasoconstriction (Budhiraja, Tuder & Hassoun, 2004). Uremic toxins can trigger chronic hemolysis, release free hemoglobin, and promote PH by scavenging nitric oxide (Billings et al., 2011). Finally, hyperparathyroidism is frequently complicated with hypercalcemia and coronary vascular calcification, thus stimulating the development of coronary artery disease, myocardial hypertrophy, and fibrosis (Amann et al., 2003; Ketteler et al., 2011). Autopsies of ESRD patients have indicated that vascular calcification is present in 40–80% of those patients (Devasahayam et al., 2020). The improvement in pulmonary vascular compliance and vascular calcification after removal of the parathyroid glands suggests that hyperparathyroidism may be a potential etiological factor for CKD-PH (Sise, Courtwright & Channick, 2013).

The therapeutic measures for CKD also bring about structural changes in the cardiovascular system and the development of PH. In CKD patients undergoing long-term hemodialysis, the creation of an AVF is a common procedure. After that, these patients show a trend towards LV dilatation and an increase in LV mass, which is positively correlated with systemic inflammation (Sahinoz et al., 2020). Specifically, they present with increases in the LV internal diameter and LV end-diastolic and end-systolic volumes, as well as cardiac dysfunction (Meinel et al., 2020). The creation of an AVF accelerates the development of left heart failure and PH, which is more likely to occur in patients complicated with heart disease or large aneurysmal AVF (Nakhoul et al., 2005). The venous shower of microbubbles from the tubing or dialyzer is unavoidable during dialysis in CKD patients. These microbubbles can obstruct the microvascular system of pulmonary arteries, potentially leading to inflammation, complement activation, ischemia, and sclerosis, thus exacerbating PH (Kosmadakis et al., 2013). A study compared PH before and after dialysis with cellulose membranes *vs*. biocompatible high-flux polysulfone dialyzers and showed a greater reduction in PH treated with biocompatible high-flux dialyzers (Kawar et al., 2013). PH was not significantly altered post-dialysis using cellulose acetate membrane, whereas synthetic and modified cellulose membranes attenuated but did not eliminate it. High-flux polysulfone filters showed a pronounced reduction in post-dialysis PH compared to cellulose acetate filters. Hence, the correlation between PH and dialysis membranes is also crucial (Kiykim et al., 2010).

In summary, factors such as substance abnormalities, comorbidities, and treatment methods of CKD are implicated in the pathogenesis or progression of PH, resulting in changes in cardiac structure and function (Yu et al., 2017). Cardiac insufficiency due to myocardial hypertrophy and fibrosis in turn contributes to the onset and worsening of PH (O’Byrne et al., 2015). CKD-PH and cardiac remodeling share common pathogenic factors and are causative of each other. Therefore, echocardiography helps to provide better clinical screening for PH in CKD patients and to monitor and manage CKD patients more comprehensively in the future. Currently, there are fewer studies on the progression of cardiac structural changes and PH in CKD patients. More studies are needed to further elucidate the specific mechanisms underlying the correlation between CKD-PH and echocardiographic parameters. In this study, we systematically integrated previous findings on the correlation between echocardiographic parameters and PH and revealed the presence of significant cardiac structural and functional alterations in CKD-PH patients, including an increase in the total volume of the cardiac chambers, thickening of the interventricular septum and interatrial septal tissues, and LV dysfunction. However, the sample of included studies is small and there are no reports of echocardiographic databases for CKD-PH patients. Currently, several representative disease-specific echocardiographic databases have been established in the field of cardiology. Given the high incidence of CKD-PH and its widespread effects on the heart, we believe that there is an urgent need to build a set of echocardiographic databases specifically for CKD-PH patients. This will enable clinicians and researchers to explore the pathophysiological mechanisms and clinical diagnosis and treatment strategies in a more in-depth and comprehensive manner, thus realizing the close connection and two-way promotion between basic research and clinical application. Based on this, we advocate that future research should focus on the establishment and utilization of echocardiographic databases of CKD-PH patients, to attract more attention and practical application. Such databases will greatly enrich our data on large samples of CKD-PH patients and thus enhance the reliability and validity of research results. Our findings could also provide practical evidence and deepen concepts in such cutting-edge areas as ‘translational clinical implications’ or ‘therapeutic echocardiographic integration’.

This study has some limitations: 24 studies were finally included, but the correlation between various echocardiographic parameters and CKD-PH was documented in only 11 studies. The number of included study samples is limited, and the echocardiographic database for CKD-PH patients has not been reported in the literature. Few of the existing articles have used either ‘multivariable logistic regression analysis’ or ‘propensity score matching’. It is expected that future studies will make greater use of these two methods to ensure that intergroup comparability and assessed relationships are not affected by other confounding factors (Ding et al., 2022; Feng & Ren, 2020; Gao et al., 2021; He et al., 2015; Lv et al., 2019; Shen et al., 2021; Wang et al., 2014a, 2014b; Yang & Bao, 2012; Yang & Bao, 2014; Zhang et al., 2020) (all the included articles are shown in 44–54 in the references).

## Conclusion

Statistical results unveil that CKD-PH is associated with left atrial enlargement, right atrial chamber enlargement, increases in LVDD and LVDS, and increased cardiac work. In CKD, increased fluid and pressure loads in the circulation may be the main reason for PH. Ultimately, the cardiac workload exacerbates decompensation until heart failure. Therefore, reducing fluid loads and increasing cardiac compensation of CKD patients may be the key therapeutic and preventive methods for reducing PH incidence. The present study incorporated many echocardiographic parameters and a substantial number of patients, so the conclusions were relatively reliable. Additional clinical studies with more patients are still needed to further support and refine the conclusions.

## Supplemental Information

10.7717/peerj.17245/supp-1 Supplemental Information 1 PRISMA checklist.

10.7717/peerj.17245/supp-2 Supplemental Information 2 Systematic Review and/or Meta-Analysis Rationale.

10.7717/peerj.17245/supp-3 Supplemental Information 3 Forest Plot for Correlation and Sensitivity Analysis and Search strategy.

## Additional Information and Declarations

### Data Availability

The following information was supplied regarding data availability: This is a systematic review/meta-analysis.
