# Supplementary material for: Meta-analysis of the correlation between pulmonary hypertension and echocardiographic parameters in patients with chronic kidney disease
Source: PeerJ. 2024 Apr 19;12:e17245. doi: 10.7717/peerj.17245 (PMC11034503; doi:10.7717/peerj.17245)
Supplement: Supplemental Information 2 [file peerj-12-17245-s002.docx]

## Systematic Review and/or Meta-Analysis Rationale

For systematic reviews / meta-analyses, authors need to provide the following information:

1.The rationale for conducting the systematic review / meta-analysis.

**Reply:** Cardiovascular disease is the main cause of death in CKD patients, and pulmonary hypertension has a high incidence in patients with cardiovascular disease, which can be as high as 50% in dialysis patients. Traditional right heart catheterization is limited in its application due to its invasiveness and high price. In contrast, echocardiography, with its low price and non-invasive characteristics, is widely used for the detection of PH in CKD patients. However, the correlation between its parameters and CKD-PH is still unknown. This META analysis was performed to better identify the presence of PH in CKD patients in the clinic and help to provide more comprehensive treatment and management of CKD patients in the future.

2.The contribution that it makes to knowledge in light of previously published related reports, including other meta-analyses and systematic reviews.

**Reply:** Previous studies have shown mixed results regarding the correlation between PH and various echocardiographic parameters in CKD patients, and there is a lack of systematic review and meta-analysis to synthesize this evidence. Our study revealed that 12 parameters were correlated with PH in CKD patients, including three categories: left heart parameters (LA, LVDD, LVPW, FS, LVEF, SV, LVMI, and LVDS), right heart parameters (RA and RV), and other parameters (IVS and PA). Except for FS and LVEF, which were negatively correlated with PH in CKD patients, the other 10 parameters were positively correlated. The parameter that was highly correlated with PH in CKD patients included LA, those that were moderately correlated included LVDD, RA, RV, LVMI, and LVDS, and those that were lowly correlated included PA, IVS, LVPW, SV, FS, and LVEF.
