# Supplementary material for: Meta-analysis of the correlation between pulmonary hypertension and echocardiographic parameters in patients with chronic kidney disease
Source: PeerJ. 2024 Apr 19;12:e17245. doi: 10.7717/peerj.17245 (PMC11034503; doi:10.7717/peerj.17245)
Supplement: Supplemental Information 3 [file peerj-12-17245-s003.docx]

**CONTENT**

1. **Forest plot for correlation and sensitivity analysis (page 1 to 8)**
2. **Funnel plot for correlation analysis (page 9 to 20)**
3. **Search strategy (page 21 to 26)**
4. **Quality evaluation form for articles (page 27)**
5. **Quality evaluation criteria (page 28 to 29)**

**Page1**

**Forest Plot for Correlation and Sensitivity Analysis**

**
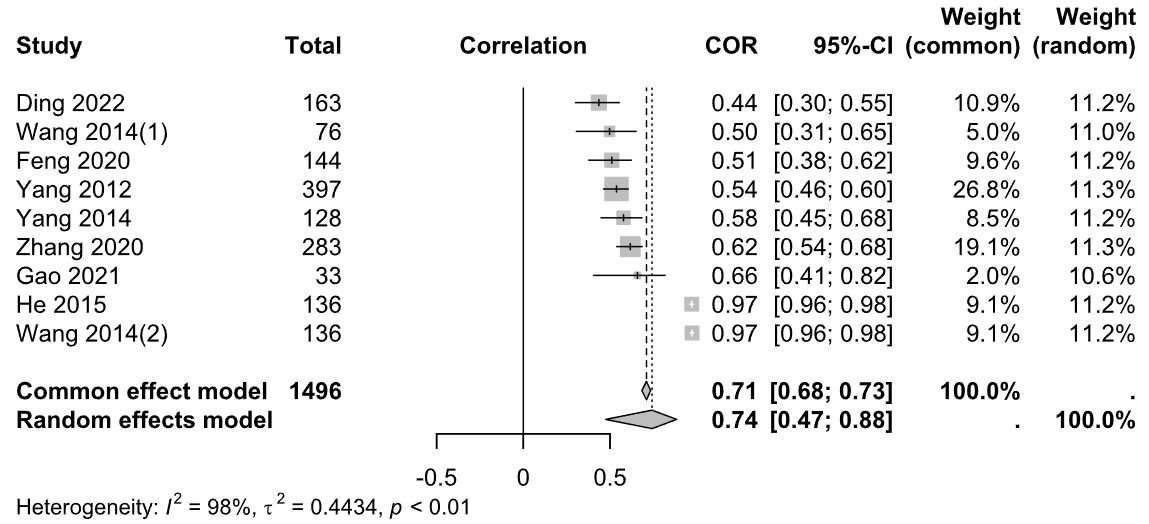
**

**Forest plot of the correlation between LA and pH in CKD patients（Fig.S1）**


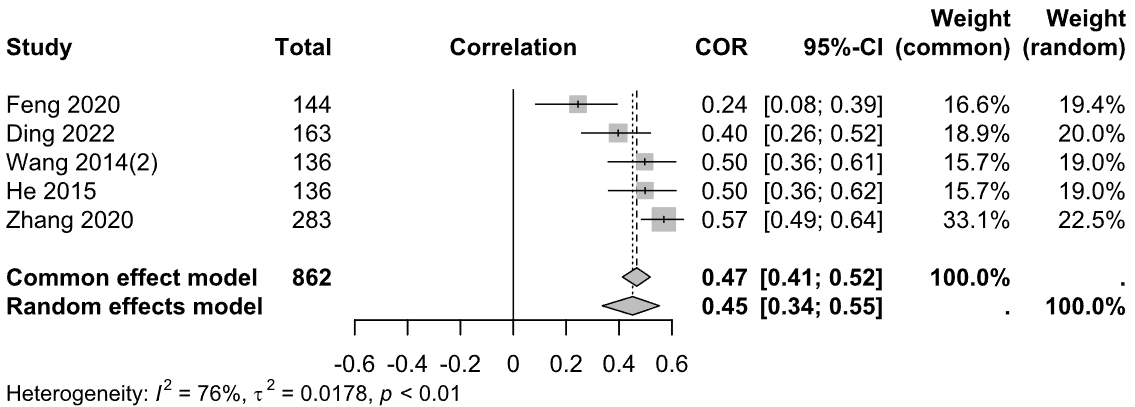


**Forest plot of the correlation between LVDD and PH in CKD patients（Fig.S2）**


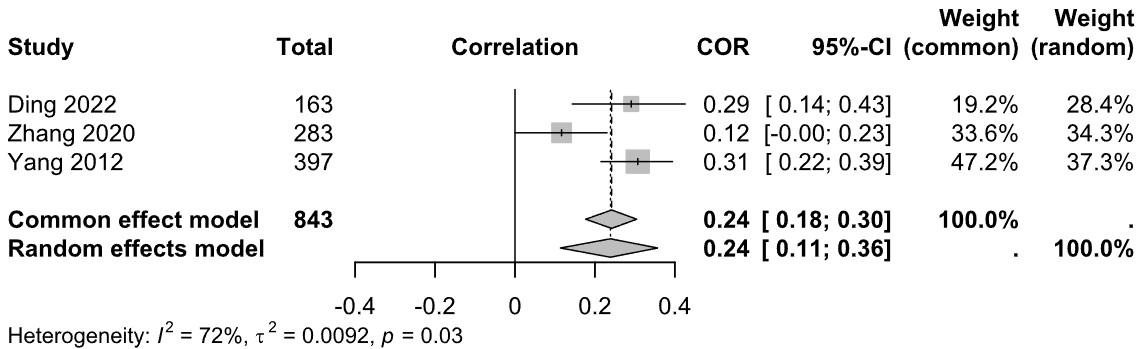


**Forest plot of the correlation between LVPW and PH in CKD patients（Fig.S3）**

**Page2**


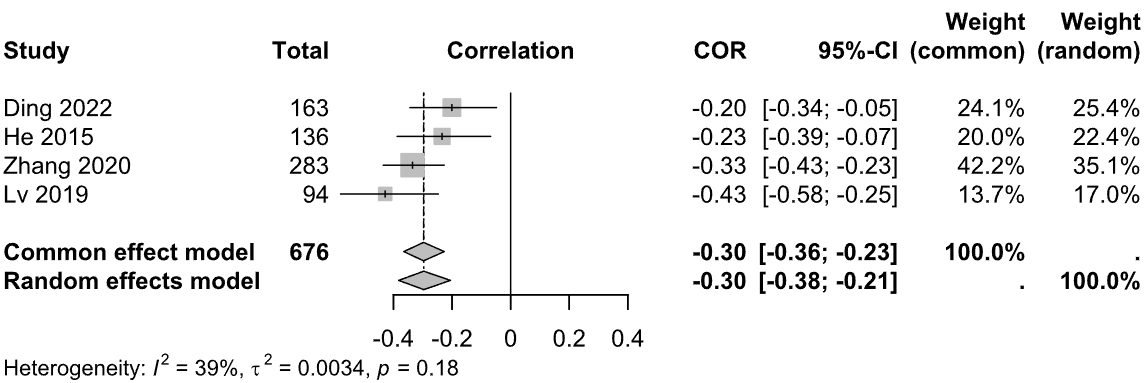


**Forest plot of the correlation between FS and PH in CKD patients（Fig.S4）**


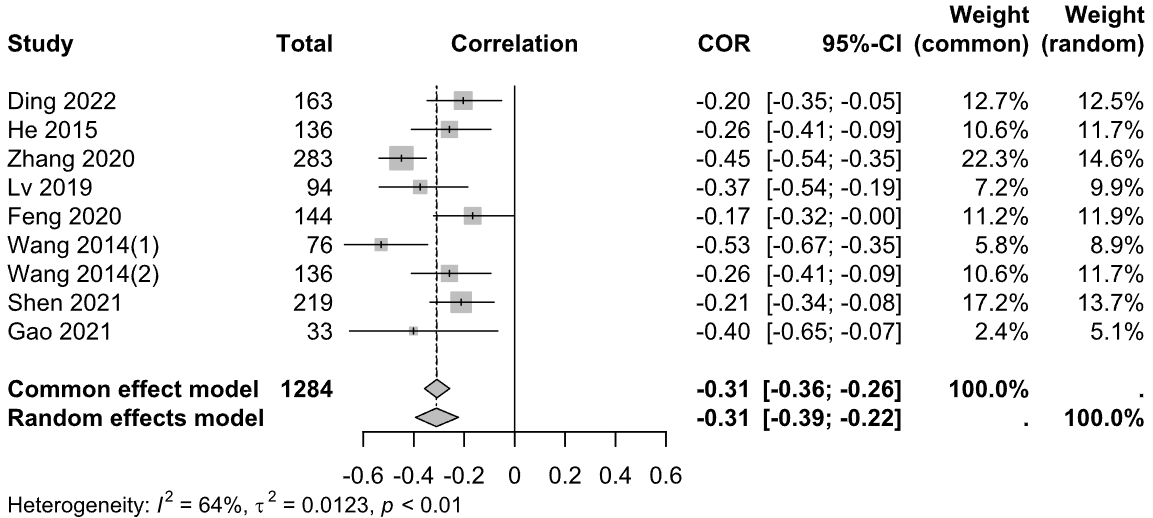


**Forest plot of the correlation between LVEF and PH in CKD patients（Fig.S5）**

**
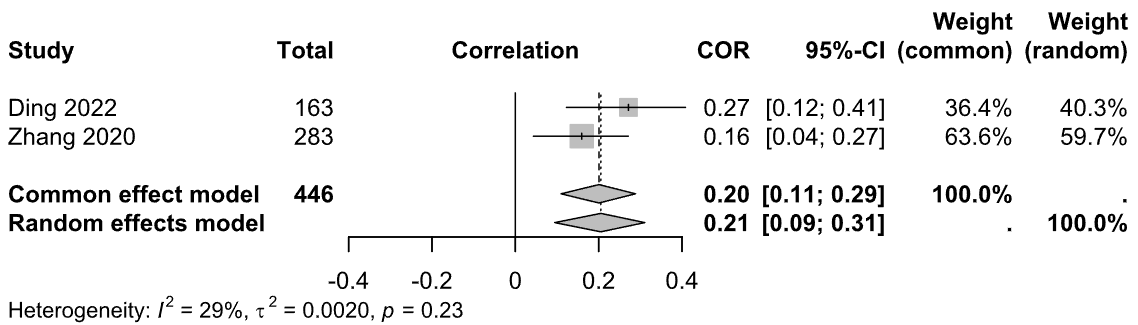
Forest plot of the correlation between SV and PH in CKD patients（Fig.S6）**

**Page3**


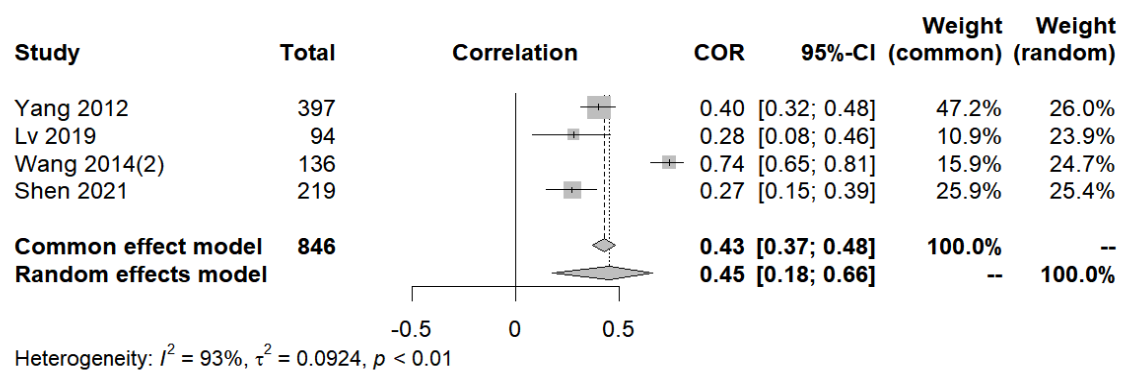


**Forest plot of the correlation between LVMI and PH in CKD patients（Fig.S7）**


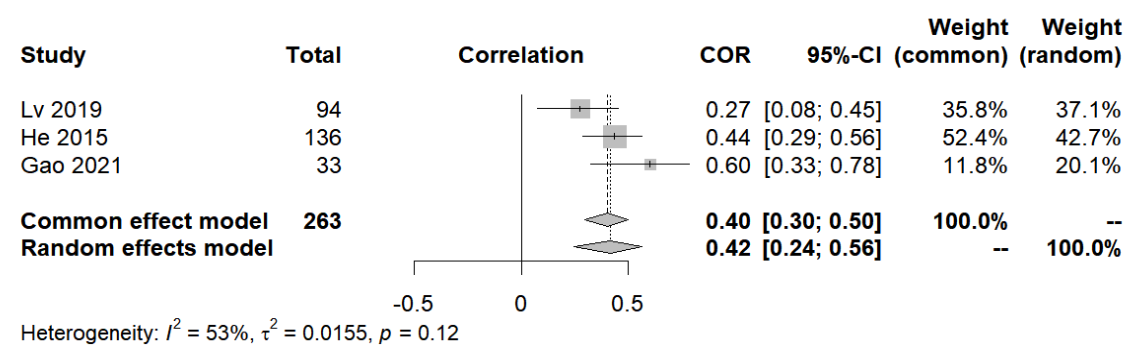


**Forest plot of the correlation between LVDS and PH in CKD patients（Fig.S8）**


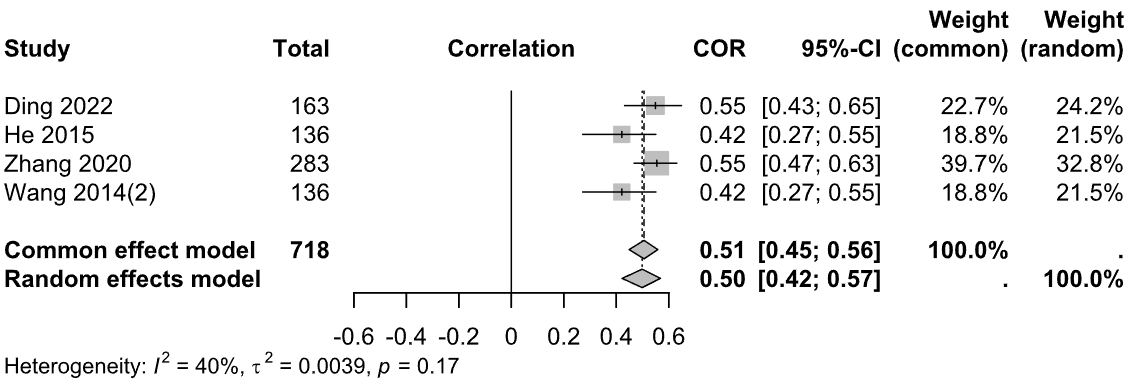


**Forest plot of the correlation between RA and PH in CKD patients（Fig.S9）**

**Page4**

**
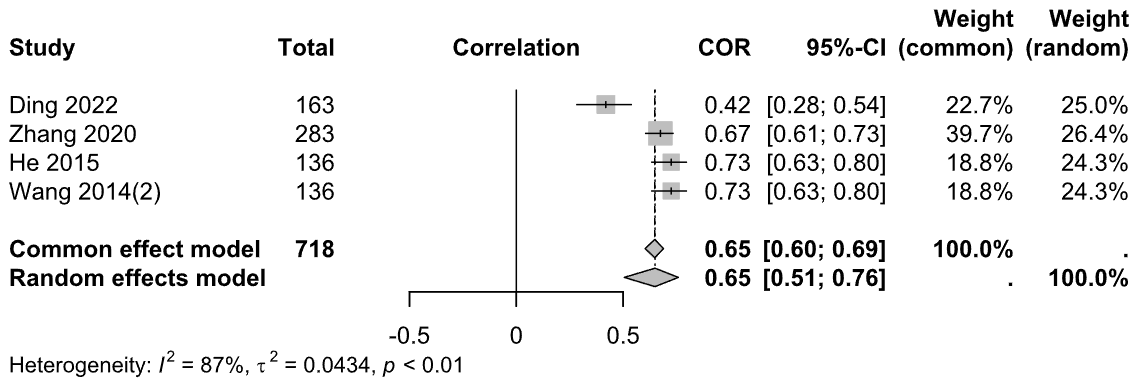
Forest plot of the correlation between RV and PH in CKD patients（Fig.S10）**


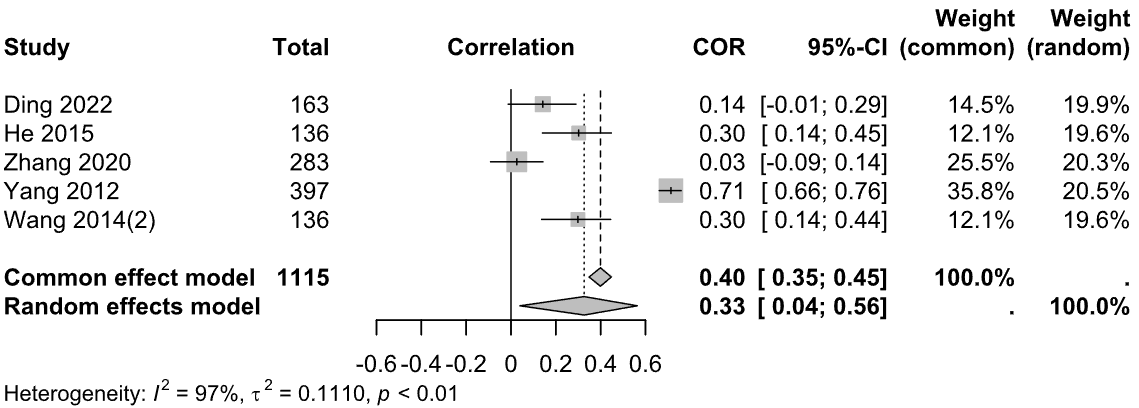


**Forest plot of the correlation between IVS and PH in CKD patients（Fig.S11）**


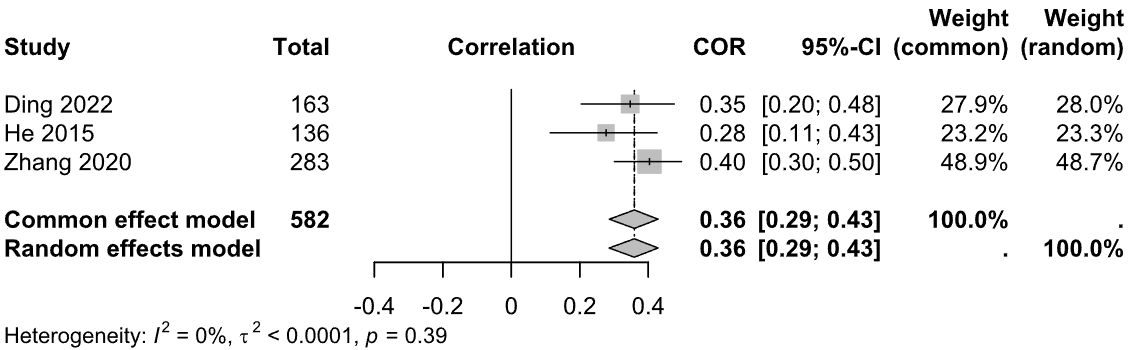


**Forest plot of the correlation between PA and PH in CKD patients（Fig.S12）**

**Page5**


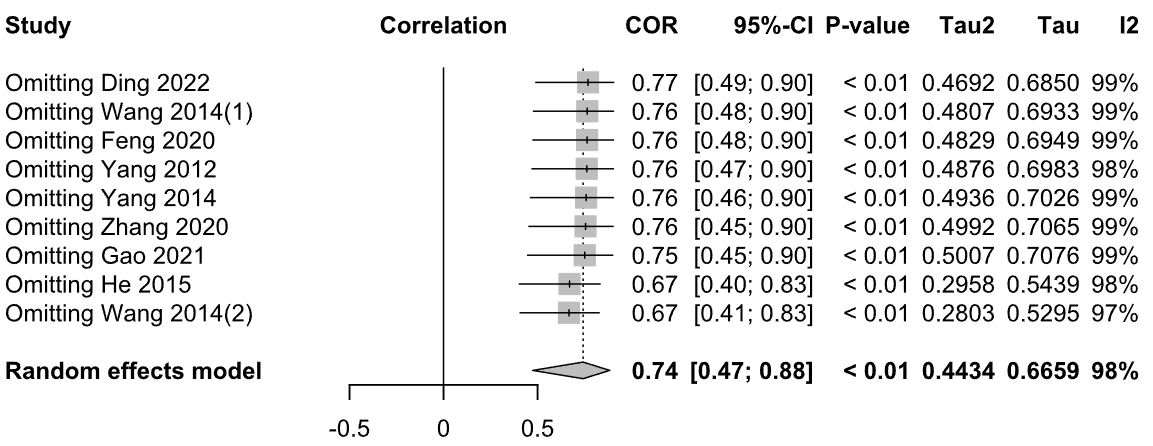


**Forest plot for sensitivity analysis of the correlation between LA and CKD patients' pH（Fig.S13）**


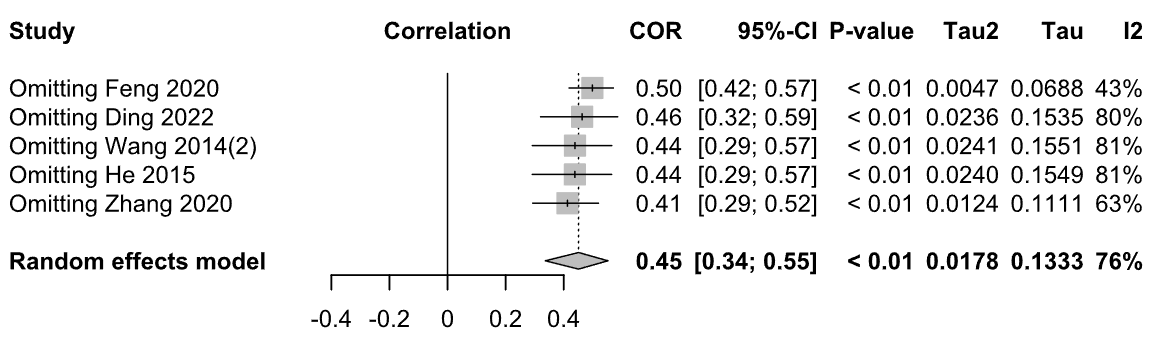


**Forest plot for sensitivity analysis of the correlation between LVDD and CKD patients' pH（Fig.S14）**


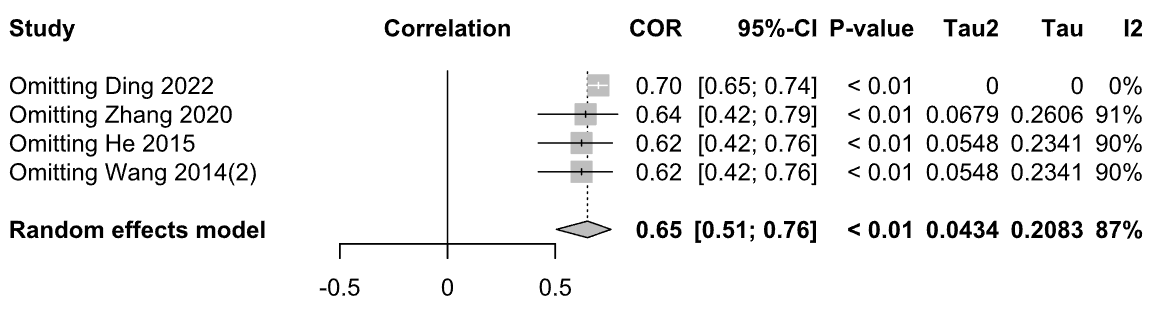


**Forest plot for sensitivity analysis of the correlation between RV and CKD patients' pH（Fig.S15）**

**Page6**


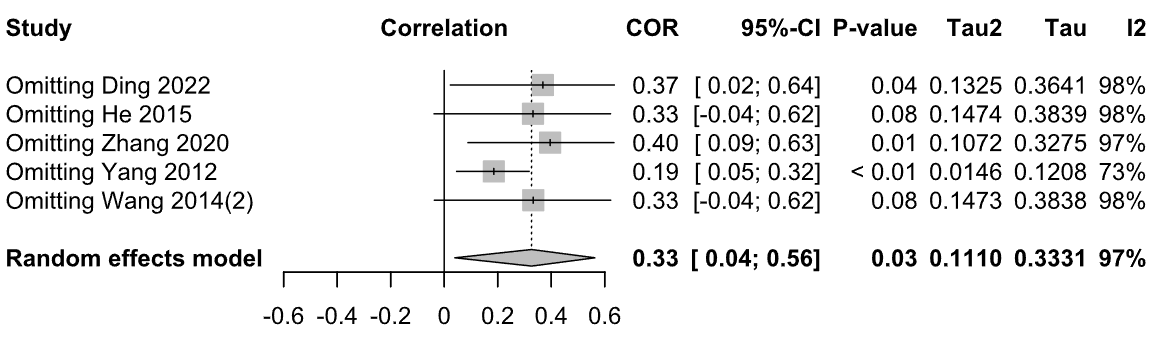


**Forest plot for sensitivity analysis of the correlation between IVS and CKD patients' pH（Fig.S16）**


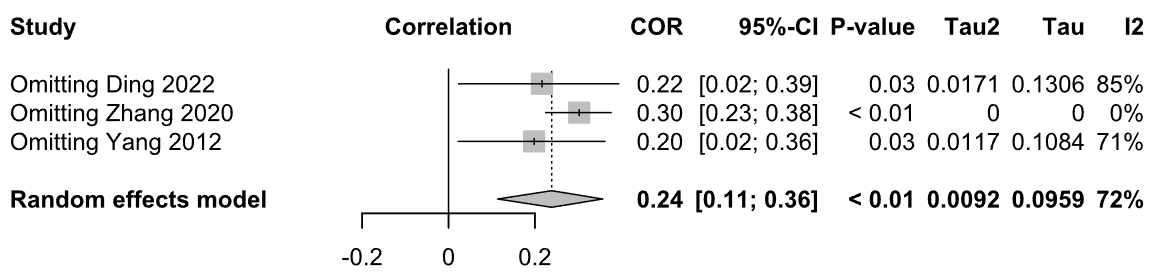


**Forest plot for sensitivity analysis of the correlation between LVPW and CKD patients' pH（Fig.S17）**


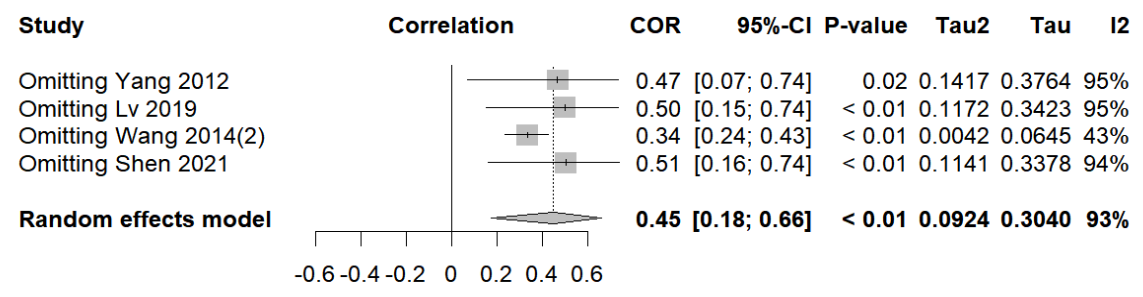


**Forest plot for sensitivity analysis of the correlation between LVMI and CKD patients' pH（Fig.S18）**

**Page7**


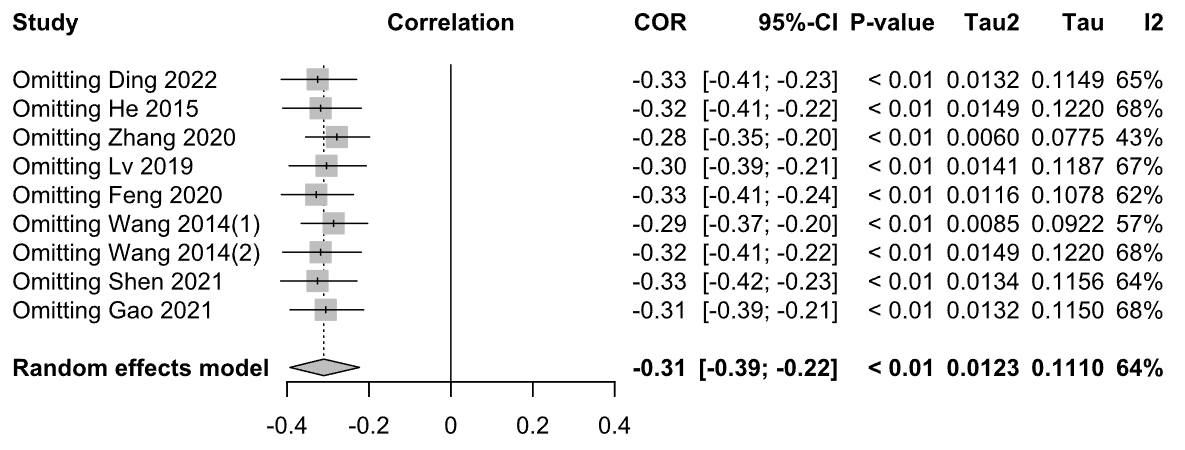


**Forest plot for sensitivity analysis of the correlation between LVEF and CKD patients' pH（Fig.S19）**

**Page8**

**Funnel plot for correlation analysis**

**
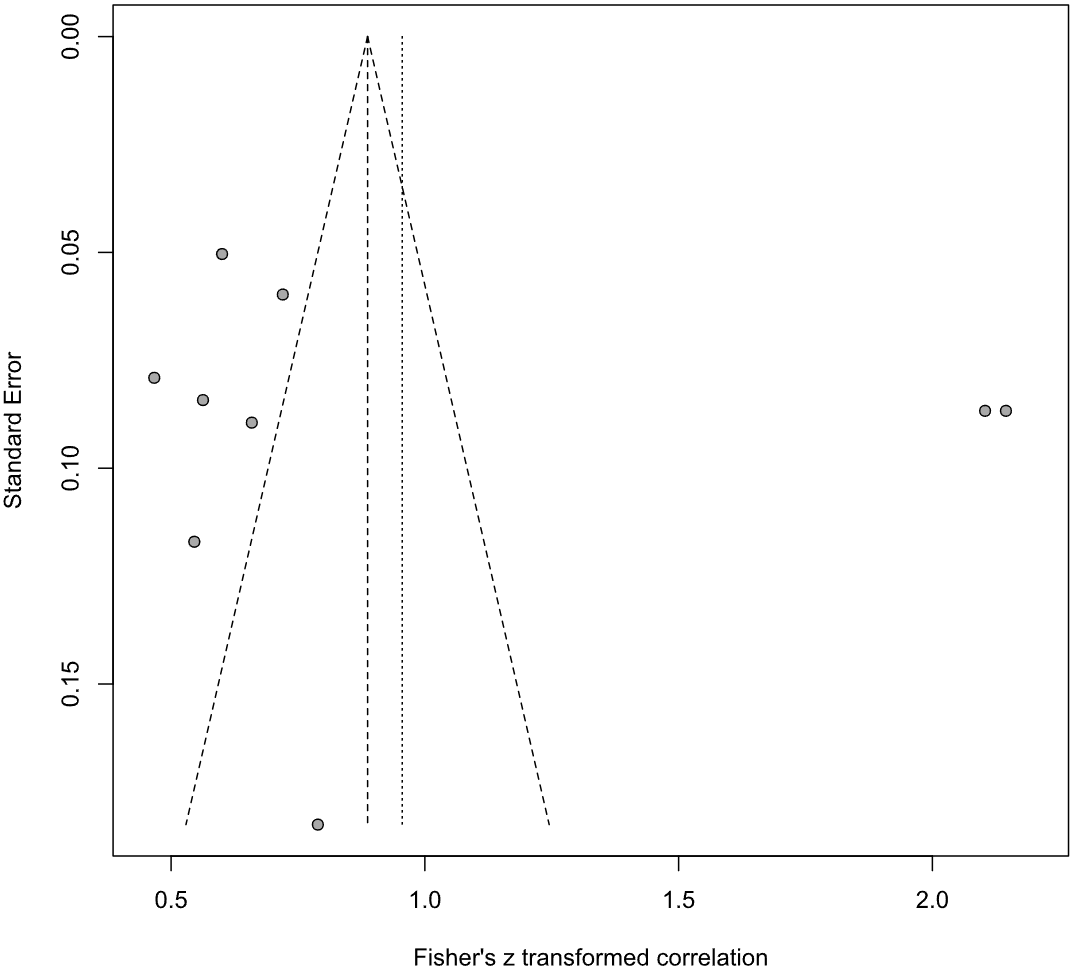
**

**Funnel plot of the correlation between LA and pH in CKD patients（Fig.S20）**

**Page9**

**
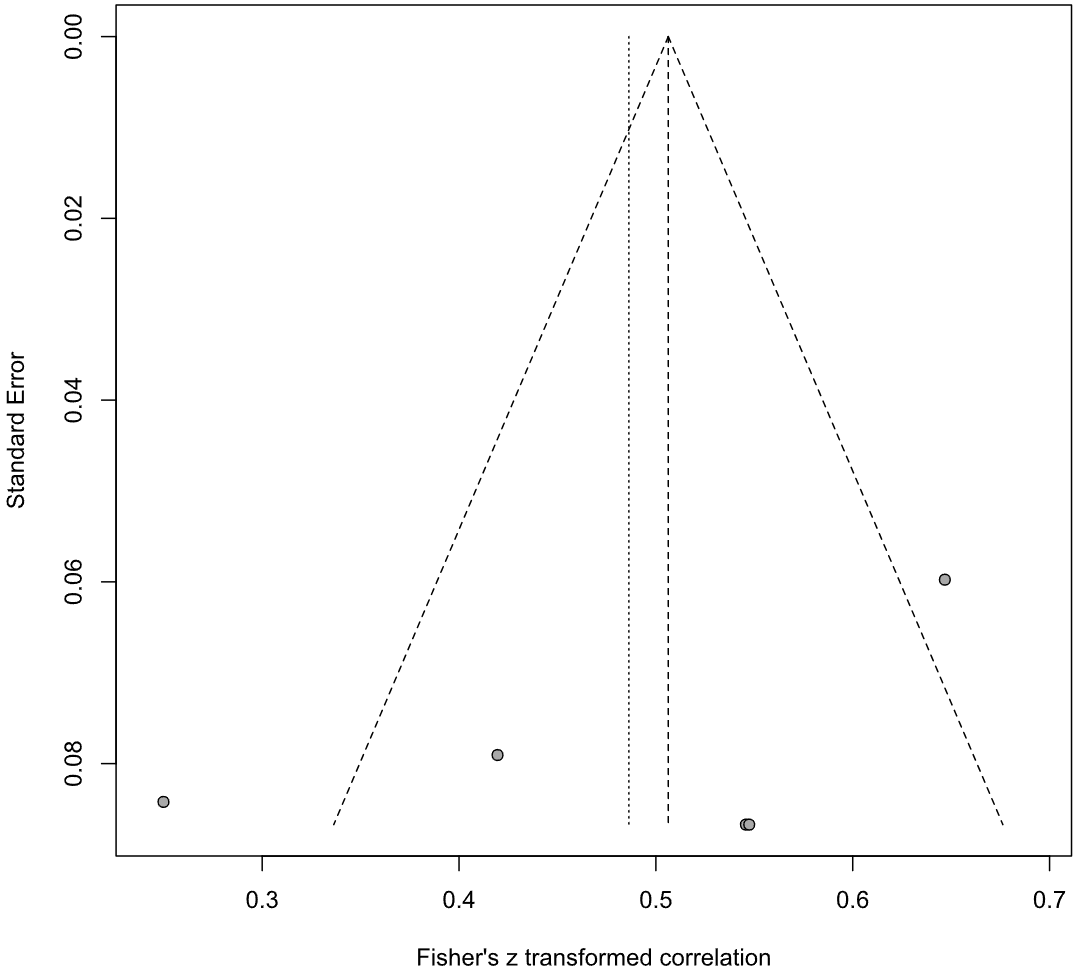
**

**Funnel plot of the correlation between LVDD and pH in CKD patients（Fig.S21）**

**Page10**

**
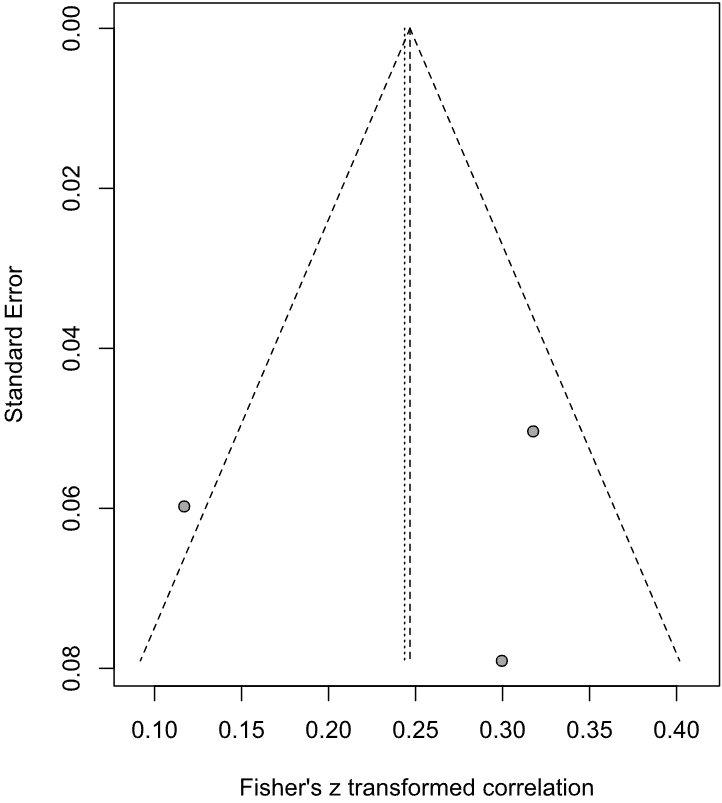
**

**Funnel plot of the correlation between LVPW and pH in CKD patients（Fig.S22）**

**Page11**

**
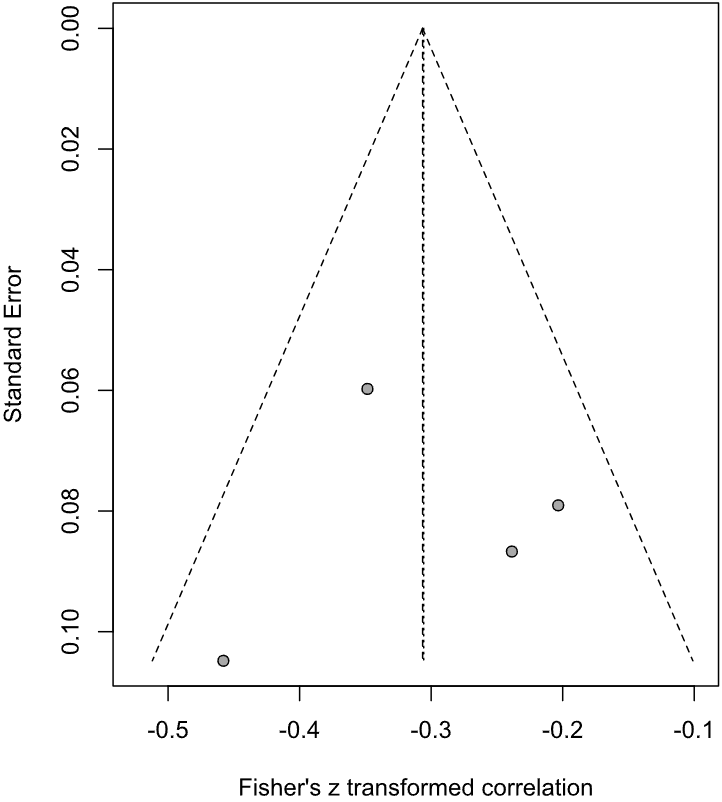
**

**Funnel plot of the correlation between FS and pH in CKD patients（Fig.S23）**

**Page12**

**
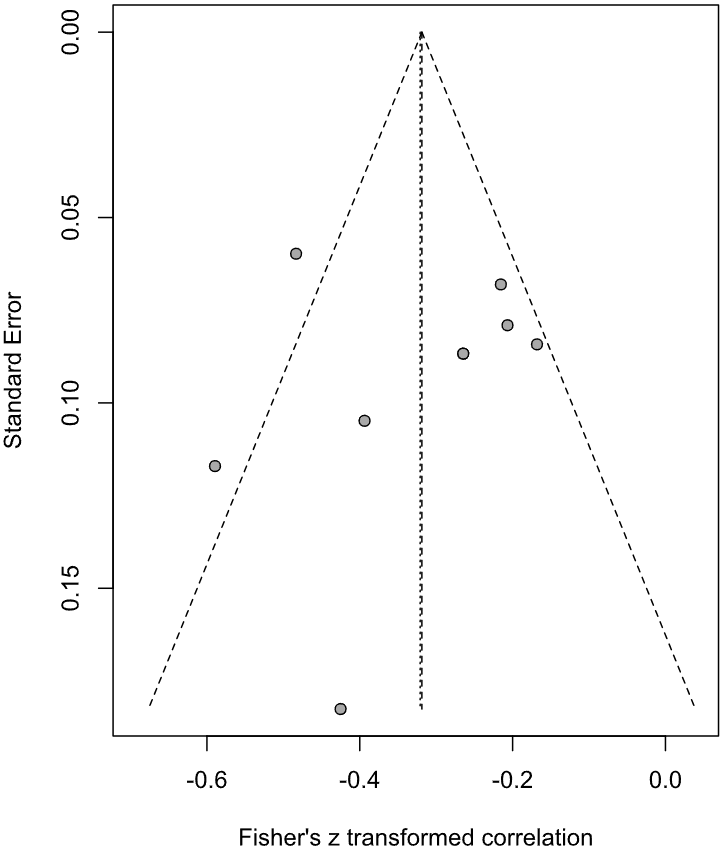
**

**Funnel plot of the correlation between LVEF and pH in CKD patients（Fig.S24）**

**Page13**

**
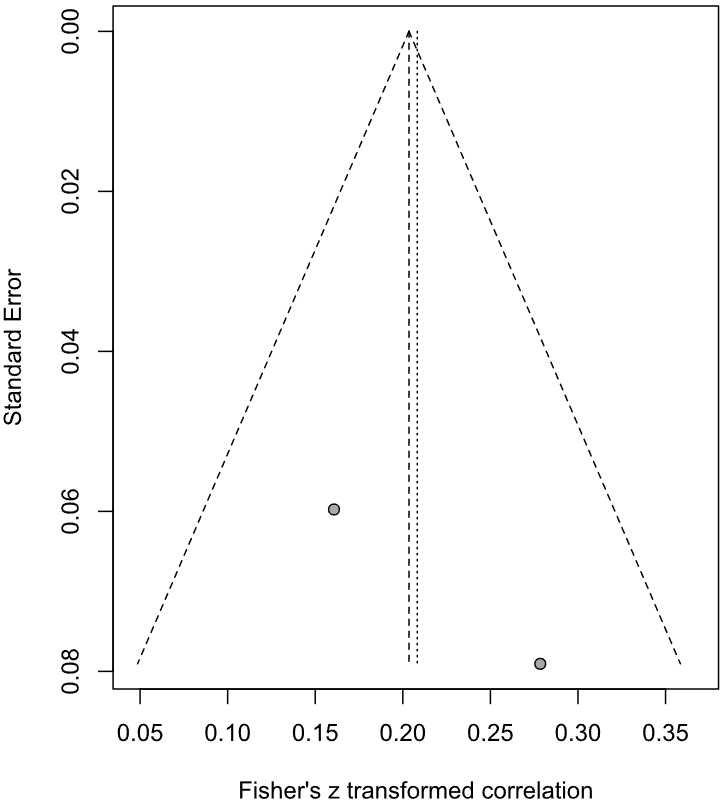
**

**Funnel plot of the correlation between SV and pH in CKD patients（Fig.S25）**

**Page14**

**
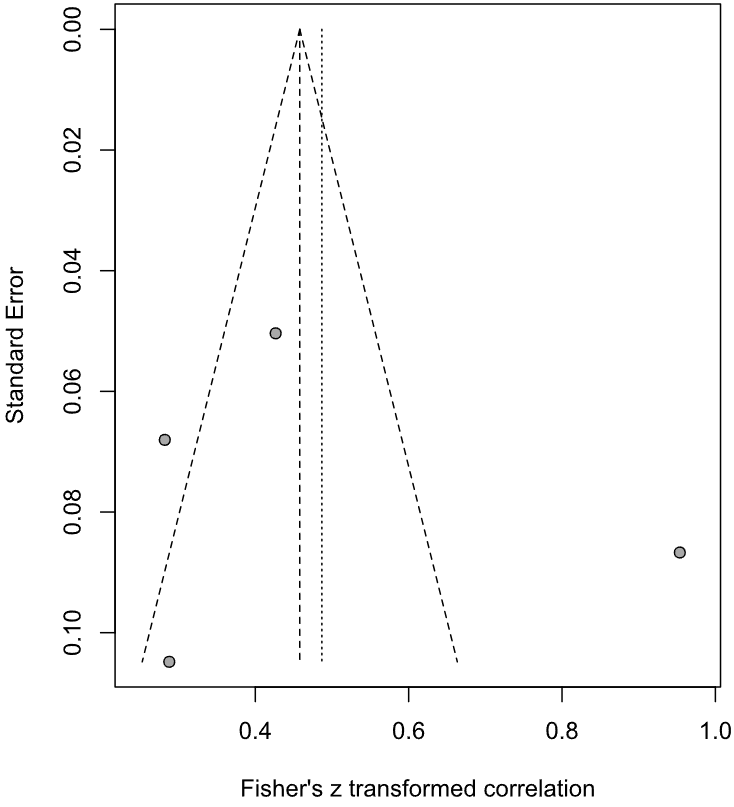
**

**Funnel plot of the correlation between LVMI and pH in CKD patients（Fig.S26）**

**Page15**

**
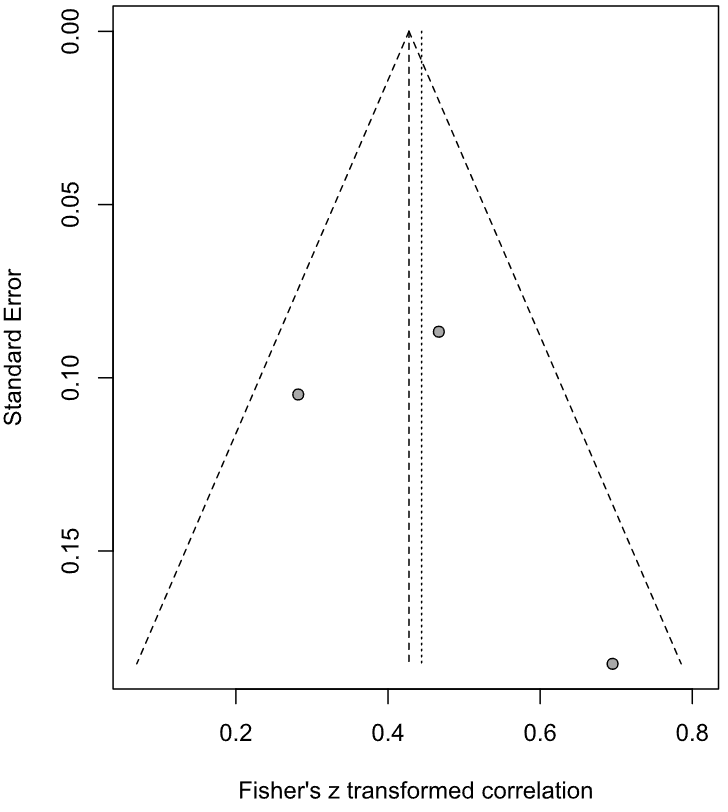
**

**Funnel plot of the correlation between LVDS and pH in CKD patients（Fig.S27）**

**Page16**

**
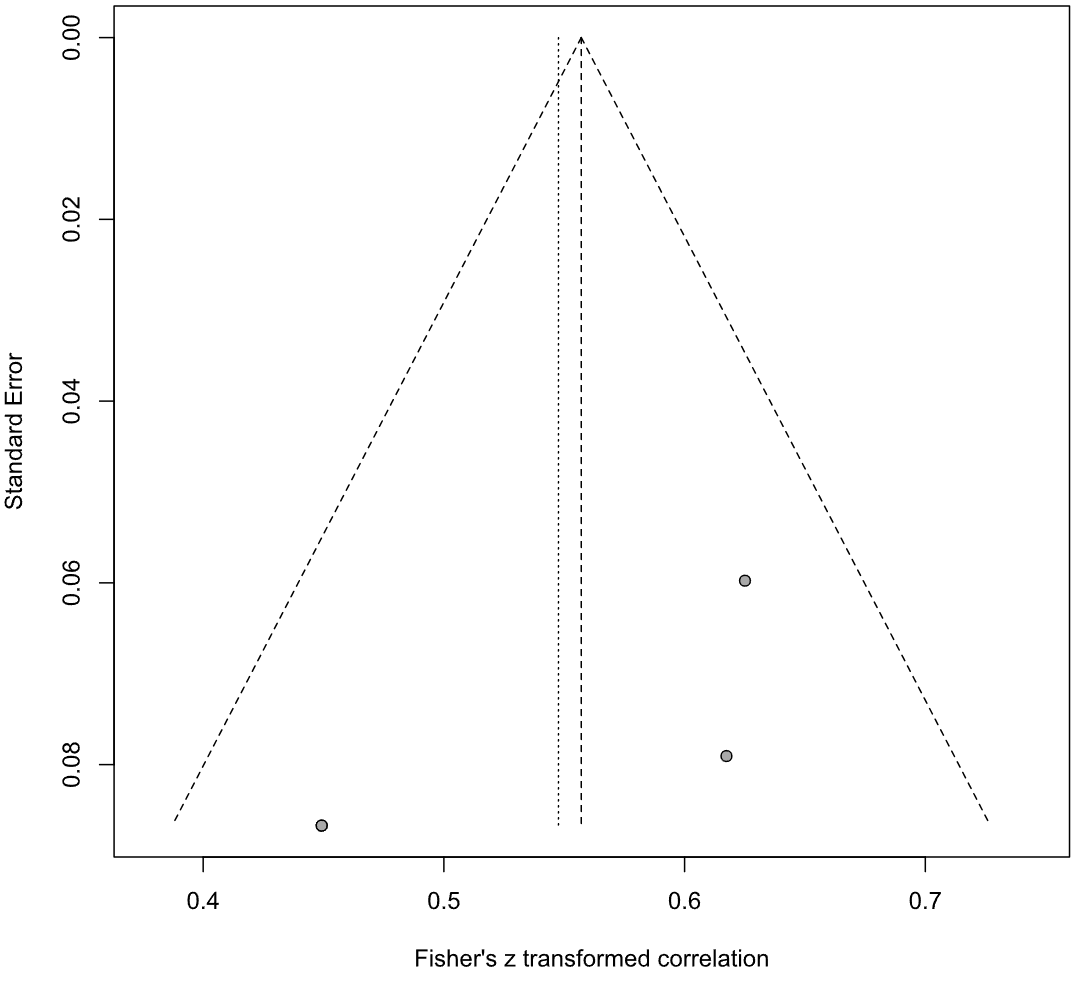
**

**Funnel plot of the correlation between RA and pH in CKD patients（Fig.S28）**

**Page17**

**
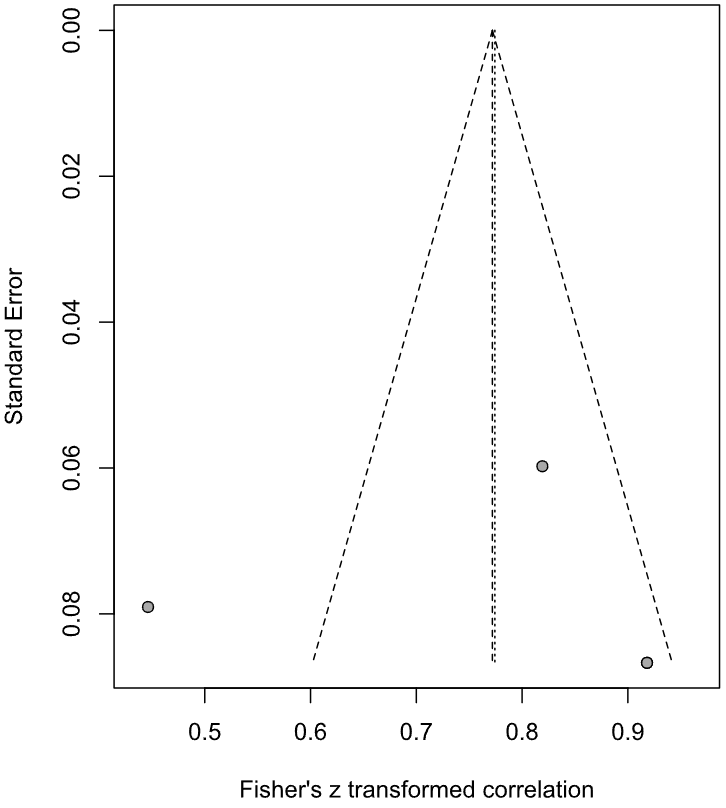
**

**Funnel plot of the correlation between RV and pH in CKD patients（Fig.S29）**

**Page18**

**
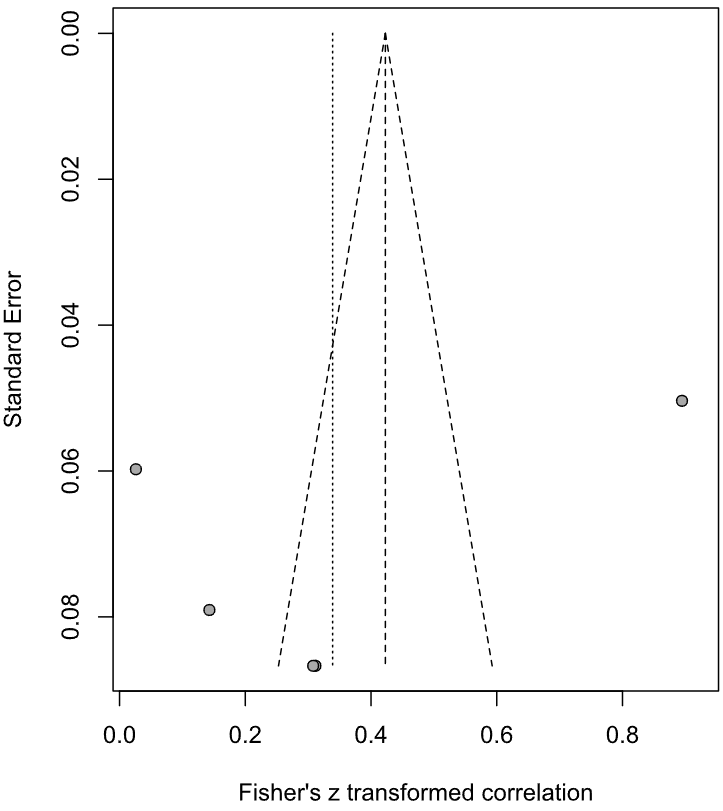
**

**Funnel plot of the correlation between IVS and pH in CKD patients（Fig.S30）**

**Page19**

**
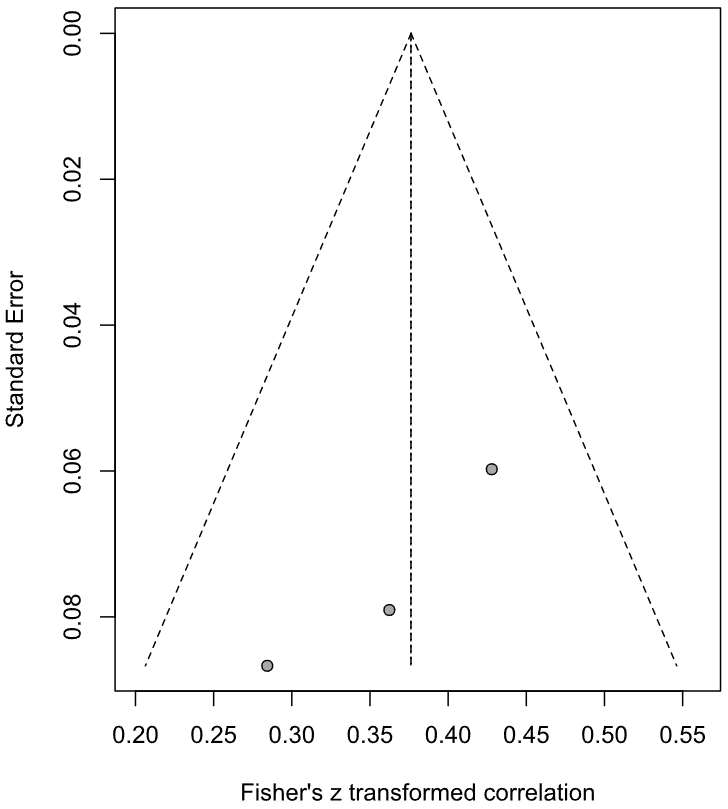
**

**Funnel plot of the correlation between PA and pH in CKD patients（Fig.S31）**

**Page20**

**Search strategy**

**PUBMED**:

“Chronic Kidney Disease*" OR "Chronic Kidney disorder" OR "Chronic Kidney Insufficiencies" OR "Chronic Kidney Insufficiency" OR "Chronic nephropathy" OR "Chronic Renal Disease*" OR "Chronic Renal failure" OR "Chronic Renal Insufficiencies" OR "Chronic Renal Insufficiency" OR "Kidney chronic failure" OR "Kidney failure, chronic" OR "Kidney function, chronic disease" OR "Chronic Kidney Failure" OR "Chronic Renal Failure" OR "End Stage Kidney Disease" OR "End stage kidney failure" OR "End Stage Renal Disease" OR "End stage renal dysfunction" OR "End Stage Renal Failure" OR "End stage renal impairment" OR "End stage renal insufficiency" OR "ESRD" OR "Stage 5 kidney disease" OR "Stage 5 renal disease" OR "Kidney Failure*" OR "Kidney Insufficiencies" OR "Kidney Insufficiency" OR "maternal kidney failure" OR "Renal Failure*" OR "Renal Insufficiencies" OR "renal insufficiency" OR "terminal kidney failure" OR "acute dialysis" OR "coil dialysis" OR "Dialyses" OR "Dialyses, Extracorporeal" OR "Dialysis, Extracorporeal" OR "Dialysis, Renal" OR "flow dialysis" OR "Hemodialyses" OR "Hemodialysis" OR "Renal Dialyses"

"Renal Insufficiency, Chronic" OR "Kidney Failure, Chronic" OR "Renal Insufficiency" OR "Renal Dialysis"

"Pulmonary Hypertension" OR "essential pulmonary hypertension" OR "familial primary pulmonary hypertension" OR "hypertensive pulmonary vascular disease" OR "idiopathic pulmonary arterial hypertension" OR "lung arterial hypertension" OR "lung artery hypertension" OR "lung hypertension" OR "primary pulmonary hypertension" OR "pulmonary arterial hypertension" OR "pulmonary artery hypertension" OR "pulmonary fixed hypertension" OR "pulmonary hypertensive disease*" OR "pulmonary hypertensive disorder*"

Pulmonary Hypertension

**Page21**

**EMBASE:**

('Chronic Kidney Disease*' OR 'Chronic Kidney disorder' OR 'Chronic Kidney Insufficiencies' OR 'Chronic Kidney Insufficiency' OR 'Chronic nephropathy' OR 'Chronic Renal Disease*' OR 'Chronic Renal failure' OR 'Chronic Renal Insufficiencies' OR 'Chronic Renal Insufficiency' OR 'Kidney chronic failure' OR 'Kidney failure, chronic' OR 'Kidney function, chronic disease' OR 'Chronic Kidney Failure' OR 'Chronic Renal Failure' OR 'End Stage Kidney Disease' OR 'End stage kidney failure' OR 'End Stage Renal Disease' OR 'End stage renal dysfunction' OR 'End Stage Renal Failure' OR 'End stage renal impairment' OR 'End stage renal insufficiency' OR 'ESRD' OR 'Stage 5 kidney disease' OR 'Stage 5 renal disease' OR 'Kidney Failure*' OR 'Kidney Insufficiencies' OR 'Kidney Insufficiency' OR 'maternal kidney failure' OR 'Renal Failure*' OR 'Renal Insufficiencies' OR 'renal insufficiency' OR 'terminal kidney failure' OR 'acute dialysis' OR 'coil dialysis' OR 'Dialyses' OR 'Dialyses, Extracorporeal' OR 'Dialysis, Extracorporeal' OR 'Dialysis, Renal' OR 'flow dialysis' OR 'Hemodialyses' OR 'Hemodialysis' OR 'Renal Dialyses'):ti,ab,kw

(‘Chronic kidney failure' or 'End-stage renal disease' or 'kidney failure' or 'dialysis')/exp

('Pulmonary Hypertension' OR 'essential pulmonary hypertension' OR 'familial primary pulmonary hypertension' OR 'hypertensive pulmonary vascular disease' OR 'idiopathic pulmonary arterial hypertension' OR 'lung arterial hypertension' OR 'lung artery hypertension' OR 'lung hypertension' OR 'primary pulmonary hypertension' OR 'pulmonary arterial hypertension' OR 'pulmonary artery hypertension' OR 'pulmonary fixed hypertension' OR 'pulmonary hypertensive disease*' OR 'pulmonary hypertensive disorder*'):ti,ab,kw

‘Pulmonary Hypertension’/exp

**Page22**

**COCHRANE:**

('Chronic Kidney Disease*' OR 'Chronic Kidney disorder' OR 'Chronic Kidney Insufficiencies' OR 'Chronic Kidney Insufficiency' OR 'Chronic nephropathy' OR 'Chronic Renal Disease*' OR 'Chronic Renal failure' OR 'Chronic Renal Insufficiencies' OR 'Chronic Renal Insufficiency' OR 'Kidney chronic failure' OR 'Kidney failure, chronic' OR 'Kidney function, chronic disease' OR 'Chronic Kidney Failure' OR 'Chronic Renal Failure' OR 'End Stage Kidney Disease' OR 'End stage kidney failure' OR 'End Stage Renal Disease' OR 'End stage renal dysfunction' OR 'End Stage Renal Failure' OR 'End stage renal impairment' OR 'End stage renal insufficiency' OR 'ESRD' OR 'Stage 5 kidney disease' OR 'Stage 5 renal disease' OR 'Kidney Failure*' OR 'Kidney Insufficiencies' OR 'Kidney Insufficiency' OR 'maternal kidney failure' OR 'Renal Failure*' OR 'Renal Insufficiencies' OR 'renal insufficiency' OR 'terminal kidney failure' OR 'acute dialysis' OR 'coil dialysis' OR 'Dialyses' OR 'Dialyses, Extracorporeal' OR 'Dialysis, Extracorporeal' OR 'Dialysis, Renal' OR 'flow dialysis' OR 'Hemodialyses' OR 'Hemodialysis' OR 'Renal Dialyses'):ti,ab,kw

‘Renal Insufficiency, Chronic' or 'Kidney Failure, Chronic' or 'Renal Insufficiency' or 'dialysis'

('Pulmonary Hypertension' OR 'essential pulmonary hypertension' OR 'familial primary pulmonary hypertension' OR 'hypertensive pulmonary vascular disease' OR 'idiopathic pulmonary arterial hypertension' OR 'lung arterial hypertension' OR 'lung artery hypertension' OR 'lung hypertension' OR 'primary pulmonary hypertension' OR 'pulmonary arterial hypertension' OR 'pulmonary artery hypertension' OR 'pulmonary fixed hypertension' OR 'pulmonary hypertensive disease*' OR 'pulmonary hypertensive disorder*'):ti,ab,kw

‘Pulmonary Hypertension’

**Page23**

**Web of science：**(Chronic Kidney Disease*) OR (Chronic Kidney disorder) OR (Chronic Kidney Insufficiencies) OR (Chronic Kidney Insufficiency) OR (Chronic nephropathy) OR (Chronic Renal Disease*) OR (Chronic Renal failure) OR (Chronic Renal Insufficiencies) OR (Chronic Renal Insufficiency) OR (Kidney chronic failure) OR (Kidney failure, chronic) OR (Kidney function, chronic disease) OR (Chronic Kidney Failure) OR (Chronic Renal Failure) OR (End Stage Kidney Disease) OR (End stage kidney failure) OR (End Stage Renal Disease) OR (End stage renal dysfunction) OR (End Stage Renal Failure) OR (End stage renal impairment) OR (End stage renal insufficiency) OR (ESRD) OR (Stage 5 kidney disease) OR (Stage 5 renal disease) OR (Kidney Failure*) OR (Kidney Insufficiencies) OR (Kidney Insufficiency) OR (maternal kidney failure) OR (Renal Failure*) OR (Renal Insufficiencies) OR (renal insufficiency) OR (terminal kidney failure) OR (acute dialysis) OR (coil dialysis) OR (Dialyses) OR (Dialyses, Extracorporeal) OR (Dialysis, Extracorporeal) OR (Dialysis, Renal) OR (flow dialysis) OR (Hemodialyses) OR (Hemodialysis) OR (Renal Dialyses)

(Pulmonary Hypertension) OR (essential pulmonary hypertension) OR (familial primary pulmonary hypertension) OR (hypertensive pulmonary vascular disease) OR (idiopathic pulmonary arterial hypertension) OR (lung arterial hypertension) OR (lung artery hypertension) OR (lung hypertension) OR (primary pulmonary hypertension) OR (pulmonary arterial hypertension) OR (pulmonary artery hypertension) OR (pulmonary fixed hypertension) OR (pulmonary hypertensive disease*) OR (pulmonary hypertensive disorder*)

(TI=(("Chronic Kidney Disease*") OR ("Chronic Kidney disorder") OR ("Chronic Kidney Insufficiencies") OR ("Chronic Kidney Insufficiency") OR ("Chronic nephropathy") OR ("Chronic Renal Disease*") OR ("Chronic Renal failure") OR ("Chronic Renal Insufficiencies") OR ("Chronic Renal Insufficiency") OR ("Kidney chronic failure") OR ("Kidney failure, chronic") OR ("Kidney function, chronic disease") OR ("Chronic Kidney Failure") OR ("Chronic Renal Failure") OR ("End Stage Kidney Disease") OR ("End stage kidney failure") OR ("End Stage Renal Disease") OR ("End stage renal dysfunction") OR ("End Stage Renal Failure") OR ("End stage renal impairment") OR ("End stage renal insufficiency") OR ("ESRD") OR ("Stage 5 kidney disease") OR ("Stage 5 renal disease") OR ("Kidney Failure*") OR ("Kidney Insufficiencies") OR ("Kidney Insufficiency") OR ("maternal kidney failure") OR ("Renal Failure*") OR ("Renal Insufficiencies") OR ("renal insufficiency") OR ("terminal kidney failure") OR ("acute dialysis") OR ("coil dialysis") OR ("Dialyses") OR ("Dialyses, Extracorporeal") OR ("Dialysis, Extracorporeal") OR ("Dialysis, Renal") OR ("flow dialysis") OR ("Hemodialyses") OR ("Hemodialysis") OR ("Renal Dialyses”)

) OR AB=(("Chronic Kidney Disease*") OR ("Chronic Kidney disorder") OR ("Chronic Kidney Insufficiencies") OR ("Chronic Kidney Insufficiency") OR ("Chronic nephropathy") OR ("Chronic Renal Disease*") OR ("Chronic Renal failure") OR ("Chronic Renal Insufficiencies") OR ("Chronic Renal Insufficiency") OR ("Kidney chronic failure") OR ("Kidney failure, chronic") OR ("Kidney function, chronic disease") OR ("Chronic Kidney Failure") OR ("Chronic Renal Failure") OR ("End Stage Kidney Disease") OR ("End stage kidney failure") OR ("End Stage Renal Disease") OR ("End stage renal dysfunction") OR ("End Stage Renal Failure") OR ("End stage renal impairment") OR ("End stage renal insufficiency") OR ("ESRD") OR ("Stage 5 kidney disease") OR ("Stage 5 renal disease") OR

**Page24**

("Kidney Failure*") OR ("Kidney Insufficiencies") OR ("Kidney Insufficiency") OR ("maternal kidney failure") OR

("Renal Failure*") OR ("Renal Insufficiencies") OR ("renal insufficiency") OR ("terminal kidney failure") OR ("acute dialysis") OR ("coil dialysis") OR ("Dialyses") OR ("Dialyses, Extracorporeal") OR ("Dialysis, Extracorporeal") OR ("Dialysis, Renal") OR ("flow dialysis") OR ("Hemodialyses") OR ("Hemodialysis") OR ("Renal Dialyses”)

) OR AK=(("Chronic Kidney Disease*") OR ("Chronic Kidney disorder") OR ("Chronic Kidney Insufficiencies") OR ("Chronic Kidney Insufficiency") OR ("Chronic nephropathy") OR ("Chronic Renal Disease*") OR ("Chronic Renal failure") OR ("Chronic Renal Insufficiencies") OR ("Chronic Renal Insufficiency") OR ("Kidney chronic failure") OR ("Kidney failure, chronic") OR ("Kidney function, chronic disease") OR ("Chronic Kidney Failure") OR ("Chronic Renal Failure") OR ("End Stage Kidney Disease") OR ("End stage kidney failure") OR ("End Stage Renal Disease") OR ("End stage renal dysfunction") OR ("End Stage Renal Failure") OR ("End stage renal impairment") OR ("End stage renal insufficiency") OR ("ESRD") OR ("Stage 5 kidney disease") OR ("Stage 5 renal disease") OR ("Kidney Failure*") OR ("Kidney Insufficiencies") OR ("Kidney Insufficiency") OR ("maternal kidney failure") OR ("Renal Failure*") OR ("Renal Insufficiencies") OR ("renal insufficiency") OR ("terminal kidney failure") OR ("acute dialysis") OR ("coil dialysis") OR ("Dialyses") OR ("Dialyses, Extracorporeal") OR ("Dialysis, Extracorporeal") OR ("Dialysis, Renal") OR ("flow dialysis") OR ("Hemodialyses") OR ("Hemodialysis") OR ("Renal Dialyses”)

)) AND (TI=(("Pulmonary Hypertension") OR ("essential pulmonary hypertension") OR ("familial primary pulmonary hypertension") OR ("hypertensive pulmonary vascular disease") OR ("idiopathic pulmonary arterial hypertension") OR ("lung arterial hypertension") OR ("lung artery hypertension") OR ("lung hypertension") OR ("primary pulmonary hypertension") OR ("pulmonary arterial hypertension") OR ("pulmonary artery hypertension") OR ("pulmonary fixed hypertension") OR ("pulmonary hypertensive disease*") OR ("pulmonary hypertensive disorder*)

) OR AB=(("Pulmonary Hypertension") OR ("essential pulmonary hypertension") OR ("familial primary pulmonary hypertension") OR ("hypertensive pulmonary vascular disease") OR ("idiopathic pulmonary arterial hypertension") OR ("lung arterial hypertension") OR ("lung artery hypertension") OR ("lung hypertension") OR ("primary pulmonary hypertension") OR ("pulmonary arterial hypertension") OR ("pulmonary artery hypertension") OR ("pulmonary fixed hypertension") OR ("pulmonary hypertensive disease*") OR ("pulmonary hypertensive disorder*)

) OR AK=(("Pulmonary Hypertension") OR ("essential pulmonary hypertension") OR ("familial primary pulmonary hypertension") OR ("hypertensive pulmonary vascular disease") OR ("idiopathic pulmonary arterial hypertension") OR ("lung arterial hypertension") OR ("lung artery hypertension") OR ("lung hypertension") OR ("primary pulmonary hypertension") OR ("pulmonary arterial hypertension") OR ("pulmonary artery hypertension") OR ("pulmonary fixed hypertension") OR ("pulmonary hypertensive disease*") OR ("pulmonary hypertensive disorder*”)

))

**Page25**

**中文：**

慢性肾脏病 OR 终末期肾病 OR 肾衰竭 OR 透析 OR 血透 OR 腹透 OR 慢性肾功能不全 OR 肾功能不全 OR 肾功能衰竭

肺动脉高压 OR 肺血管高压

**Page26**

**Table S1 Quality Evaluation Form for Articles**

| Study ID | #1 | #2 | #3 | #4 | #5 | #6 | #7 | #8 | #9 | #10 | #11 | #12 | Overall rating |
| --- | --- | --- | --- | --- | --- | --- | --- | --- | --- | --- | --- | --- | --- |
| Ding 2022 | YES | YES | YES | YES | YES | YES | YES | YES | YES | YES | NO | YES | GOOD |
| Yang 2014 | YES | YES | YES | YES | YES | YES | YES | YES | YES | YES | NO | YES | GOOD |
| He 2015 | YES | YES | YES | YES | YES | YES | YES | YES | YES | YES | NO | YES | GOOD |
| Zhang 2020 | YES | YES | YES | YES | YES | YES | YES | YES | YES | YES | NO | YES | GOOD |
| Yang 2012 | YES | YES | YES | YES | YES | YES | YES | YES | YES | YES | NO | YES | GOOD |
| Lv 2019 | YES | YES | YES | YES | YES | YES | YES | YES | YES | YES | NO | YES | GOOD |
| Feng 2020 | YES | YES | YES | YES | YES | YES | YES | YES | YES | YES | NO | YES | GOOD |
| Wang 2014(1) | YES | YES | YES | YES | YES | YES | YES | YES | YES | YES | NO | YES | GOOD |
| Wang 2014(2) | YES | YES | YES | YES | YES | YES | YES | YES | YES | YES | NO | YES | GOOD |
| Shen 2021 | YES | YES | YES | YES | YES | YES | YES | YES | YES | YES | NO | YES | GOOD |
| Gao 2021 | YES | YES | YES | YES | YES | YES | YES | YES | YES | YES | NO | YES | GOOD |

**Page27**

**Quality Evaluation Criteria**

1. Was the research question or objective in this paper clearly stated and appropriate?
2. Was the study population clearly specified and defined?
3. Did the authors include a sample size justification?
4. Were controls selected or recruited from the same or similar population that gave rise to the cases (including the same timeframe)?
5. Were the definitions, inclusion and exclusion criteria, algorithms or processes used to identify or select cases and controls valid, reliable, and implemented consistently across all study participants?
6. Were the cases clearly defined and differentiated from controls?
7. If less than 100 percent of eligible cases and/or controls were selected for the study, were the cases and/or controls randomly selected from those eligible?
8. Was there use of concurrent controls?

**Page28**

1. Were the investigators able to confirm that the exposure/risk occurred prior to the development of the condition or event that

defined a participant as a case?

1. Were the measures of exposure/risk clearly defined, valid, reliable, and implemented consistently (including the same time period) across all study participants?
2. Were the assessors of exposure/risk blinded to the case or control status of participants?
3. Were key potential confounding variables measured and adjusted statistically in the analyses? If matching was used, did the investigators account for matching during study analysis?

Note:The above twelve items are the NIH's criteria for assessing the literature. Each item consists of one question, and the overall evaluation is categorized as good, fair, or poor, and the specific evaluation results are shown in Table S1, from Quality Assessment of Case-Control Studies in Study Quality Assessment Tools | NHLBI, NIH. Assessment of Case-Control Studies.

**Page29**
